# Distinct transmission sites within a synapse for strengthening and homeostasis

**DOI:** 10.1126/sciadv.ads5750

**Published:** 2025-04-11

**Authors:** Yue Yang, Man Ho Wong, Xiaojie Huang, Delia N. Chiu, Yu-Zhang Liu, Vishnu Prabakaran, Amna Imran, Elisa Panzeri, Yixuan Chen, Paloma Huguet, Alexander Kunisky, Jonathan Ho, Yan Dong, Brett C. Carter, Weifeng Xu, Oliver M. Schlüter

**Affiliations:** ^1^ Department of Neuroscience, University of Pittsburgh, Pittsburgh, PA, USA.; ^2^ European Neuroscience Institute Göttingen (ENI-G), ENI-G, a Joint Initiative of the University Medical Center Göttingen and the Max Planck Institute for Multidisciplinary Sciences, Göttingen, Germany.; ^3^ Department of Bioengineering, University of Pittsburgh, Pittsburgh, PA, USA.; ^4^ Department of Psychiatry and Psychotherapy, University Medical Center Göttingen, Göttingen, Germany.

## Abstract

At synapses, miniature synaptic transmission forms the basic unit of evoked transmission, thought to use one canonical transmission site. Two general types of synaptic plasticity, associative plasticity to change synaptic weights and homeostatic plasticity to maintain an excitatory balance, are so far thought to be expressed at individual canonical sites in principal neurons of the cortex. Here, we report two separate types of transmission sites, termed silenceable and idle-able, each participating distinctly in evoked or miniature transmission in the mouse visual cortex. Both sites operated with a postsynaptic binary mode with different unitary sizes and mechanisms. During postnatal development, silenceable sites were unsilenced by associative plasticity with α-amino-3-hydroxy-5-methyl-4-isoxazolepropionate (AMPA)-receptor incorporation, increasing evoked transmission. Concurrently, miniature transmission remained constant, where AMPA-receptor state changes balanced unsilencing with increased idling at idle-able sites. Thus, individual cortical spine synapses mediated two parallel, interacting types of transmission, which predominantly contributed to either associative or homeostatic plasticity.

## Teaser

Glutamatergic synapses operate with two parallel transmission sites that encode memories versus maintain homeostasis.

## INTRODUCTION

Information transfer between neurons relies on the registration of quantal neurotransmitter packets by postsynaptic receptors. These packets are released when action potentials trigger the fusion of synaptic vesicles. The quantal content refers to the number of these vesicle fusions, and it is generally believed that postsynaptic receptors reliably detect and transform this quantity into postsynaptic currents. In addition, the quantal size is determined by the number of postsynaptic receptors at the synapse (*1*, *2*). As such, the generally homogeneous postsynaptic response (quantal) size of miniature excitatory postsynaptic currents (mEPSCs) may represent the basic units, the multitude of which constitutes the evoked EPSCs (*1*, *3*, *4*). However, this model homogenizes the potential variation of postsynaptic receptors, whose states determine how effectively neurotransmitters released by the presynaptic neuron are detected.

In glutamatergic synapses of principal neurons in the mammalian cortex, quantal sizes have a high variance with a positively skewed amplitude distribution (*5*, *6*) that is caused at least partially by variations in released glutamate at single synapses (*7*). Furthermore, a silent synaptic state exists where synapses do not respond with α-amino-3-hydroxy-5-methyl-4-isoxazolepropionate (AMPA)–type glutamate receptors (AMPARs) and respond only with *N*-methyl-d-aspartate (NMDA)–type glutamate receptors (NMDARs) (*8*–*10*). Consequently, the quantal content of evoked AMPAR-mediated synaptic transmission is determined by both the number of presynaptic vesicle fusion events and the availability of AMPAR-responsive transmission sites to detect and transform them into postsynaptic currents.

Silent synapses and their maturation provide a unique cellular model to explore AMPAR-responsive transmission sites. They are abundant during critical periods of mammalian cortex development, where they contribute to neural network refinement and their maturation contributes to closing critical periods (*11*–*13*). During neural network refinement, silent synapses can undergo two key processes: They can mature and become responsive by incorporating synaptic AMPARs, which is mediated by NMDAR-dependent long-term synaptic potentiation, a form of associative plasticity, or they may be pruned (*8*, *9*, *14*, *15*). Depending on the fate of silent synapses, the strength of excitatory drive is expected to change differently.

A unitary connection, defined as a connection of a single axon with a single postsynaptic neuron, is formed between layer 4 (L4) and L2/3 pyramidal neurons, with an average of four synapses per connected pair (*16*). When silent synapses mature, it is anticipated to increase the registration of quantal content in evoked unitary connections (*9*, *11*, *17*). Conversely, a decrease in quantal content and unitary transmission may occur if pruning of mature synapses containing AMPARs dominates the refinement process (*18*). No measured change would be expected if the pruning of mature synapses and silent synapse maturation balance each other. Supporting this equilibration theory of silent synapse maturation and pruning neuron-wide, the frequency of miniature synaptic transmission remains constant during critical periods, and no compensatory changes in quantal size have been observed (*15*, *19*). However, whether the excitatory drive at unitary connections and, thus, evoked transmission changes during critical periods remains unknown.

In the classical view, miniature synaptic transmission and evoked synaptic transmission occur at the same transmission sites (*1*, *20*). However, at glutamatergic synapses, miniature synaptic transmission and evoked synaptic transmission may involve distinct synaptic vesicle pools and receptor clusters (*21*–*23*). Consistent with this notion, AMPARs assemble into distinct nanoclusters at the postsynapse that may align with presynaptic release sites (*24*, *25*). It is unknown whether these clusters constitute independent transmission sites and whether their plasticity is regulated differently to maintain the constant frequency of miniature transmission during critical periods in cortical principal neuron synapses while evoked transmission matures via associative plasticity.

Here, we discovered two types of transmission sites at individual synapses: “silenceable sites,” which regulated evoked synaptic transmission, and “idle-able sites,” which regulated the responsiveness of AMPARs through a long-lasting unresponsive state, termed “idle,” primarily contributing to miniature synaptic transmission. Both types of sites could transition between on and off states, but they did so through different mechanisms—synaptic AMPAR incorporation at silenceable sites and AMPAR idling at idle-able transmission sites. During critical periods, previously silent synaptic transmission became responsive at silenceable transmission sites, leading to a developmental increase in evoked synaptic transmission. Concurrently, at idle-able transmission sites, idling of transmission increased. We conducted simulations of two models of transmission site configurations—one with distinct separable transmission sites at different spines and the other with transmission involving both types of sites at the same spines. Both models aligned with the observed increase in miniature synaptic transmission upon pharmacological unidling. However, they predicted different outcomes regarding the magnitude and success of responses when glutamate application activated all AMPARs of the synapse/spine. We tested those predictions using two-photon glutamate uncaging on single spine heads, revealing a mixed configuration of distinct transmission sites at single spines. Together, we uncovered two parallel transmission mechanisms at excitatory synapses in cortical principal neurons. These mechanisms were responsible for regulating mEPSC homeostasis through synapse idling plasticity at the postsynapse and respond functionally to the increased responses of quantal content at silenceable transmission sites during critical periods.

## RESULTS

### The developmental trajectories of evoked synaptic transmission versus miniature synaptic transmission were disconnected

In the classical uniform model of synaptic transmission, the same synaptic vesicles and postsynaptic receptors contribute to miniature synaptic transmission as well as evoked synaptic transmission such that glutamate from a synaptic vesicle that fuses at any location of the active zone can reach any AMPAR at the postsynaptic side (fig. S1) (*1*, *20*, *26*). A postsynaptically silent synapse without AMPARs for both miniature transmission and evoked transmission would only respond to synaptic glutamate after unsilencing.

Accumulating evidence indicates that the uniform model to describe transmission at cortical glutamatergic synapses of principal neurons is insufficient (*21*, *27*). Heterogeneous molecular machines for synaptic vesicle exocytosis might have specialized for different forms of transmission, leading to synapses preferring miniature transmission versus evoked transmission (*22*). Alternatively, they might constitute separable transmission sites at a single synapse (fig. S1). Such a mixed synapse configuration could allow differential regulation of miniature synaptic transmission and evoked synaptic transmission at a single synapse, e.g., for synapse strengthening versus homeostasis.

**Fig. 1.**
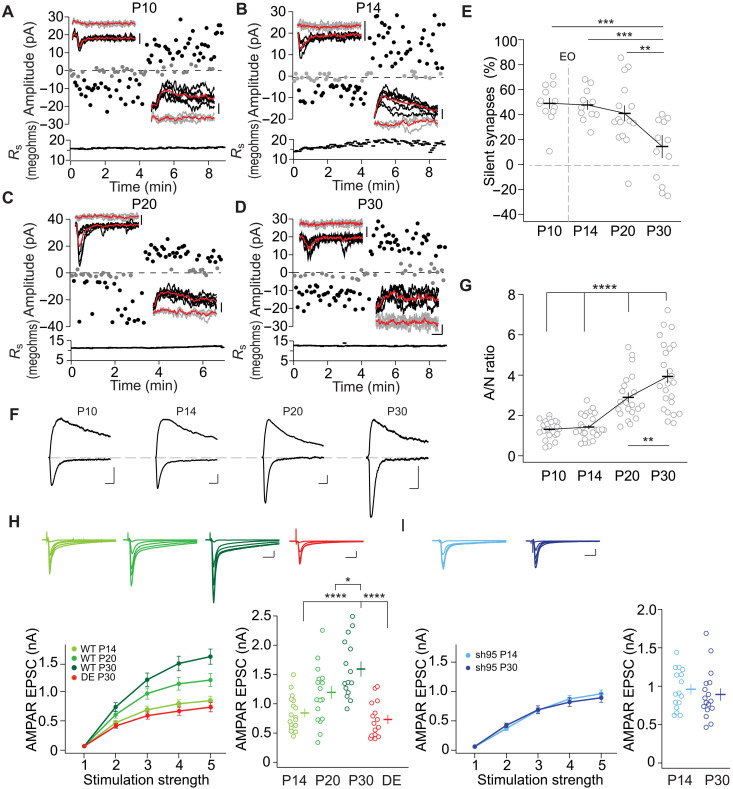
Developmental increase in AMPAR-mediated synaptic drive with silent synapse maturation. (**A** to **D**) Sample traces of EPSCs from V1 L2/3 pyramidal neurons by using minimal stimulation. The peak values of AMPAR EPSCs were recorded at *V*_*h*_ = −60 mV (downward deflection), and composite glutamate receptor EPSCs were recorded at *V*_*h*_ = +40 mV (upward deflection). Transmission successes or failures are depicted in black or gray, respectively. Averaged failures and successes are depicted as red traces. The time course of the series resistance (*R*_s_) is plotted (bottom). Scale bars, 10 ms and 10 pA. (**E**) Summary graphs of the % of silent synapses (black bars for mean values and gray circles for single neurons). The time point of eye opening (EO) is depicted as a dotted line. P10 (12/7), P14 (11/4), P20 (15/7), and P30 (12/7). *F*_3,46_ = 8.551, *P* < 0.01; P10 versus P30: ***P_adj_ < 0.001; P14 versus P30: ***P_adj_ < 0.001; P20 versus P30: **P_adj_ < 0.01. (**F**) Sample traces of EPSCs were recorded at *V*_*h*_ = −60 mV and at *V*_*h*_ = +40 mV. Scale bar, 10 ms and 20 pA. (**G**) Summary graphs of the A/N ratio. P10 (23/7), P14 (26/6), P20 (21/6), and P30 (24/6). *F*_3,90_ = 33.25, *P* < 0.0001; P10 versus P20 or P30: ****P_adj_ < 0.0001; P14 versus P20 or P30: ****P_adj_ < 0.0001; P20 versus P30: **P_adj_ < 0.01. (**H** and **I**) Input-output relationships for AMPAR EPSCs in WT with normal visual experience or dark exposure (P14-P30, DE; H) and sh95 (I) expressing neurons at indicated ages (color coded). Sample traces for EPSCs at five different stimulation intensities (top panels) and summary graphs (bottom left panels). The amplitude of the plateaued EPSC at the fifth stimulation strength is plotted against the different experimental conditions with the means ± SEM and the individual values (bottom right panel). (H) WT: P14 (17/3), P20 (17/3), P30 (15/3), and DE (15/3). *F*_3,60_ = 13.66, *P* < 0.0001; P14 versus P30: ****P_adj_ < 0.0001; P20 versus P30: *P_adj_ < 0.05; P30 versus DE: ****P_adj_ < 0.0001; P14 versus DE: *P*_adj_ > 0.99. (I) sh95: P14 (16/3) and P20 (17/3). *T*_31_ = 0.67, *P* = 0.51.

To start to gain insight into different regulations of miniature transmission and evoked transmission, we compared the developmental trajectory of either form of transmission. To determine the impact of silent synapse maturation on the overall excitatory synaptic drive onto principal neurons, we investigated the evoked synaptic transmission properties in L2/3 pyramidal neurons of the mouse visual cortex (see schematic in fig. S2A). Using failure analysis with minimal stimulation of the L4-to-L2/3 synaptic pathway, we assessed the fraction (%) of silent synapses between P10 (before eye opening), P14 (after eye opening), P20 (the beginning of the critical period), and P30 (toward the end of the critical period). The % of silent synapses was determined by the relationship of the failure rate at holding potential (*V*_*h*_) values of −60 mV and +40 mV (see Materials and Methods for the relationship equation). At the *V*_*h*_ of −60 mV, the failure rate depends on presynaptic failures and the responsiveness of AMPARs as NMDARs are blocked by Mg^2+^. At the *V*_*h*_ of +40 mV with the same stimulation strength, the failure rate depends only on presynaptic transmission failures as both AMPARs and NMDARs respond to released glutamate. Thus, a higher failure rate at *V*_*h*_ = −60 mV was indicative of a lower responsiveness of AMPARs compared to NMDARs, thus the presence of AMPAR silent synapses (Fig. 1, A to D) (*9*). Consistent with our previous results (*11*, *12*), the % of silent synapses was ~50% at P10 and P14 and decreased to <20% at P30 (Fig. 1E). Notably, the significant drop in the % of silent synapses occurred during the critical period from P20 to P30.

To further validate our model of silent synapse estimation, we simulated the minimal stimulation procedure on the basis of published synaptic parameters (see Materials and Methods for details and fig. S2) (*11*, *16*, *28*). We used the binomial model of synaptic vesicle fusion with three to five synapses in a unitary connection and % of silent synapses at P16 of 0.5 ± 0.15 and at P30 of 0.1 ± 0.15. We used a range of synaptic release probabilities between 0.3 and 0.5 on the basis of results from L2/3 pyramidal neurons of the somatosensory cortex with *P*_r_ = 0.46 (fig. S2A) (*28*). The simulation resulted in failures and activation of varying numbers of synapses (fig. S2, B and C). However, when the release probability (*P*_r_) was ~0.5, the failure rate was low and, for some simulations, failures were absent, which did not allow the calculation of the % of silent synapses. Notably, when we chose a *P*_r_ higher than 0.5, simulations without failures became more prevalent. Thus, for connections with on average four synapses, the *P*_r_ appears to be below 0.5 for transmission failures to occur at unitary connections, which we used as the upper limit of the *P*_r_ in the simulations. With the *P*_r_ between 0.3 and 0.5, when simulating 20 neurons at P16 and P30, the % of silent synapses was different (fig. S2D), and the distribution of the % of silent synapses of individual simulated neurons mimicked the distribution of values of our experimental neurons (Fig. 1E and fig. S2D), indicating that the model described our experimental preparation.

In parallel, we examined AMPAR- and NMDAR-mediated EPSCs, functional readouts of two main ionotropic glutamate receptor subtypes that may undergo development-dependent changes implicated in the assessment of silent synapses (*12*). The amplitude of AMPAR EPSCs was measured at a *V*_*h*_ = −60 mV. The NMDAR EPSC amplitude was measured at a *V*_*h*_ = +40 mV and by measuring the current 60 ms after the peak when the contribution of AMPARs to the composite EPSC is minimal (Fig. 1F) (*29*). On P30, the AMPAR-to-NMDAR EPSC amplitude (A/N) ratio has increased approximately threefold during development (Fig. 1G). This result is consistent with maturation and, thus, decreased % of silent synapses at this developmental stage. However, compared to the developmental time course of % of silent synapses, the A/N ratio already had a steep increase from P14 to P20, indicating that while the relative amount of AMPARs in synapses increased during development, changes in NMDARs might also occur. As the switch of NMDAR subunits occurs during the first three postnatal weeks (*30*, *31*), we estimated the change in synaptic NMDAR currents. For these estimates, we measured the current 60 ms after the peak of the EPSCs at a *V*_*h*_ = +40 mV from our minimal stimulation results (Fig. 1, B, C, and E). The current at P20 was smaller than that at P14 [P14: 21.3 ± 3.2 pA versus P20: 17.8 ± 2.4 pA; *P* < 0.001, Kolmogorov-Smirnov (KS) test], indicating a developmental decrease in the synaptic NMDAR currents and consistent with an additional developmental effect of NMDARs to the A/N ratio. Notably, during development, the contribution of the GluN2A subunit over the GluN2B subunit increases (*32*). Given the faster time course of GluN2A-containing NMDARs versus GluN2B-containing NMDARs, part of the reduction in NMDAR currents might be due to the subunit switch. In short, the functional outcome of developmental plasticity in the gain or wane of excitatory drive onto pyramidal neurons remained unclear.

To discern the direct impact of developmental plasticity on AMPAR-mediated excitatory drive, we quantified the overall strength of evoked AMPAR-mediated synaptic transmission at P14, P20, and P30 by examining the input-output curve of AMPAR EPSCs. We adjusted the initial stimulation strength for each neuron to obtain an initial EPSC of ~50 pA at a *V*_*h*_ = −60 mV (Fig. 1H). We then increased the stimulation strength by first doubling it and further increasing it by the same amount to record AMPAR EPSCs with altogether five levels of stimulation strength. The steepness of the input-output curve increased during development in L2/3 neurons of wild-type (WT) mice approaching a plateau (Fig. 1H). To test whether the developmental increase was visual experience dependent, we analyzed mice that were dark exposed from P14 until P30 (*15*). The input-output curve at P30 of the dark-exposed mice was like that of P14 mice, indicating that the developmental increase was dependent on visual experience. The lack of visual experience prevents silent synapse maturation (*11*, *15*, *33*). Thus, our results were consistent with an increase in excitatory drive onto pyramidal neurons because of silent synapse maturation by unsilencing during critical periods.

To test its dependence on silent synapse unsilencing further, we examined the same input-output curve in slices from postsynaptic density protein 95 (PSD-95)–deficient neurons. We expressed a short hairpin RNA (shRNA) against PSD-95 (sh95) with an adeno-associated viral vector (AAV) in the visual cortex of newborn mice. The reduced protein levels of PSD-95 impair silent synapse maturational unsilencing, and the % of silent synapses remains at ~50% throughout development and adulthood (*11*, *12*, *34*). Notably, this is a 100% penetrant phenotype because, at eye opening, PSD-95 protein levels are low in the visual cortex (*11*–*13*). The input-output curves at P14 and P30 were similar in PSD-95–deficient neurons, reaching a similar plateau (Fig. 1I). Together with the lack of increase during dark exposure, our results indicated that the critical period–associated increase in the input-output curve in WT mice was mediated by silent synapse unsilencing. Thus, the excitatory drive onto L2/3 pyramidal neurons increased during critical periods and this increase is at least partially mediated by the increase in the detection of quantal content of unitary connections after silent synapse unsilencing (*11*).

**Fig. 2.**
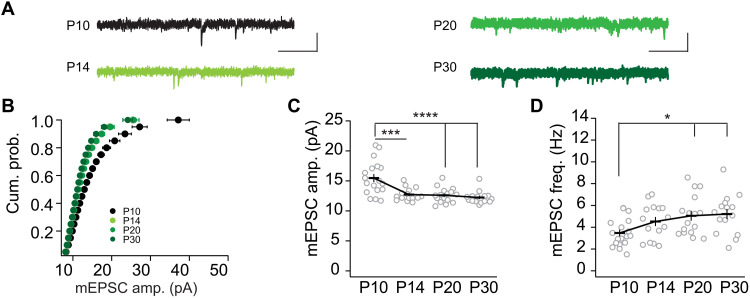
Homeostasis of mEPSCs during development after eye opening. (**A**) Sample traces for AMPAR-mediated mEPSC recorded at indicated ages (color coded). Scale bar, 200 ms and 20 pA. (**B**) Cumulative probability plots of AMPAR mEPSC amplitude. KS test: ***P < 0.001. Summary graphs of mEPSC amplitude (**C**) and frequency (**D**) dependent on postnatal (P) age (means as a black bar with SEM and average of single neurons as a gray circle). P10 (18/4), P14 (15/4), P20 (16/5), and P30 (15/4). (C) *F*_3,60_ = 12.12, *P* < 0.0001; P10 versus P14: ***P_adj_ < 0.001; P10 versus P20 or P30: ****P_adj_ < 0.0001. (D) *F*_3,60_ = 3.899, *P* < 0.05; P10 versus P20 or P30: *P_adj_ < 0.05; P14 versus P20 or P30: *P*_adj_ > 0.99.

The frequency of miniature synaptic transmission depends on primed synaptic vesicle fusogenicity, as well as the number of postsynaptically responsive transmission sites (*2*, *35*). We recorded mEPSCs in L2/3 pyramidal neurons through P10 to P30 (Fig. 2A). The cumulative probability plot of mEPSC amplitudes indicated that the curves at P14 and older were left shifted for the different amplitude bins in comparison to that at P10 (Fig. 2B). These shifts resulted in lower mean amplitudes, thus quantal sizes for mEPSCs at P14, P20, and P30 compared to P10, whereas the mean amplitudes at P14 to P30 were alike (Fig. 2C). The mEPSC frequency increased after eye opening with a difference between P10 versus P20 and P10 versus P30 (Fig. 2D), while the frequency remained constant after P14. Thus, eye opening was correlated with a decrease in amplitude and an increase in the frequency of mEPSCs. In contrast, after eye opening and throughout the critical period, mEPSC frequencies and amplitudes remained constant. This lack of developmental change despite changes in neural network configurations indicated a homeostatic regulation (*15*, *19*). Furthermore, the dissociation of the changes of evoked transmission and miniature transmission might indicate a scenario that involves distinct transmission sites as illustrated in the mixed model (fig. S1).

Spine pruning counterbalances the increase in AMPAR-mediated transmission because of silent synapse unsilencing, contributing to the equilibrium of overall AMPAR-mediated synaptic transmission (*15*). However, this mechanism cannot explain the conflict between the increased excitatory drive of evoked synaptic transmission and constant miniature synaptic transmission. To gain further insight, we assessed dendritic spines, postsynaptic structures whose density predicts the overall excitatory synapse density (but excludes shaft synapses). Using Alexa 568–based imaging of L2/3 pyramidal neuron basal dendrites (*36*), we observed that the spine density increased more than twofold from P10 to P20 and decreased by ~20% until P30 (fig. S3, A and B). Thus, as reported earlier (*15*), the spine density decreased along the proceeding of critical periods. The stark increase in spine density from P10 to P20 was likely due to the profound synaptogenesis and subsequent maturation of silent synapses (Fig. 1), which outweighs the potential spine pruning. From P20 to P30, there was a net loss of spines despite a further increase in excitatory drive (Fig. 1H). This ~20% loss was smaller than the decrease in the % of silent synapses of ~60% (from 50% at P14 to 20% at P30), resulting in a calculated net increase in excitatory drive. Thus, the decrease in the % of silent synapses was a combined consequence of synapse pruning and silent synapse unsilencing (*15*). Notably, we observed a net gain in synaptic weights at the remaining connections (Fig. 1H) but no increase in mEPSC frequency (Fig. 2D).

### Desensitization inhibitors restored unresponsive transmission sites

Blocking AMPAR desensitization pharmacologically has been reported to activate dormant transmission sites (*3*, *37*, *38*). It was initially proposed that the AMPAR desensitization blocker cyclothiazide (CTZ) exerted its effect by increasing presynaptic release probability (*3*, *37*, *38*). However, the large effect size of CTZ does not align well with the reported presynaptic CTZ mechanisms (*39*), indicating additional undiscovered effects of CTZ. To effectively detect transmission, both presynaptic vesicle release and postsynaptic receptive sites need to operate together. Thus, a postsynaptic site that either lacks AMPARs or contains only unresponsive, e.g., desensitized, AMPARs does not detect transmission and is functionally silent. If these two distinct postsynaptic sites existed, CTZ could help to experimentally restore the unresponsive AMPARs in the postsynapse. Like the unsilencing of silent synapses (*11*, *15*, *35*), restoring transmission of unresponsive sites would lead to an increase in mEPSC frequency if an entire transmission site was previously unresponsive. Notably, such a mechanism has so far only been observed when synapses were activated repeatedly with short interstimulus intervals but not for basal synaptic transmission.

**Fig. 3.**
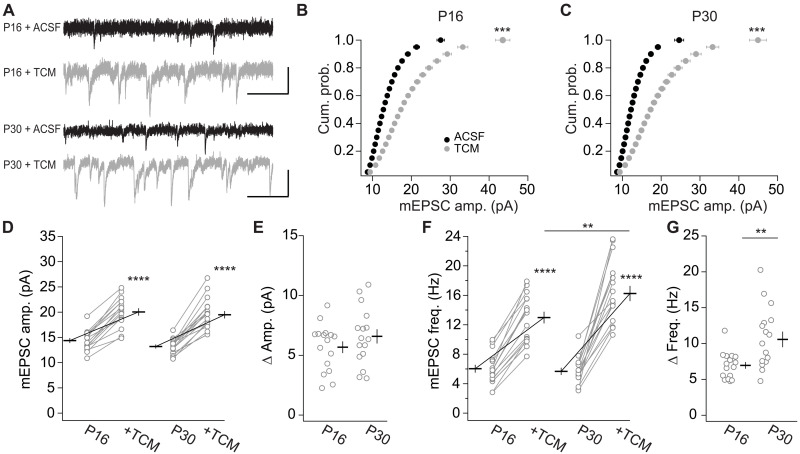
Transmission sites are restored by the desensitization blocker TCM. (**A**) Sample traces for AMPAR-mediated mEPSCs recorded in V1 L2/3 neurons from P16 and P30 mice before (ACSF) and after TCM application. Scale bar, 200 ms and 20 pA. (**B** and **C**) Cumulative probability graph of AMPAR mEPSC amplitude from P16 (B) and P30 (C). KS test: ***P < 0.001. Effect of TCM on mEPSC amplitude (**D**), amplitude increase (Δ*Amp*). (**E**), frequency (**F**), and frequency increase (Δ*Freq*.) (**G**) in WT mice at P16 and P30. P16 (16/9) and P30 (16/9). (D) *F*_30,30_ = 3.965, *P* < 0.001; ACSF versus TCM: *F*_1,30_ = 601.2, ****P < 0.0001; P16 versus P30: *F*_1,30_ = 0.791, *P* = 0.38; interaction: *F*_1,30_ = 1.360, *P* = 0.25. (E) *T*_30_ = 1.166, *P* = 0.25. (F) *F*_30,30_ = 2.318; ACSF versus TCM: *F*_1,30_ = 237.1, ****P < 0.0001; P16 versus P30: *F*_1,30_ = 3.65, *P* = 0.066; interaction: *F*_1,30_ = 11.58; **P < 0.01; P16 TCM versus P30 TCM: *P*_adj_ < 0.01. (G) *T*_30_ = 3.403, **P < 0.01.

We tested whether such a mechanism contributed to homeostatically adjusting mEPSC frequencies to remain constant after eye opening. We recorded mEPSCs from L2/3 pyramidal neurons at P16 and P30. We assessed mEPSC parameters first in standard artificial cerebrospinal fluid (ACSF) and then ~8 min after wash-in of 500 μM trichlormethiazide (TCM; Fig. 3A). We first used TCM instead of CTZ as both block AMPAR desensitization, but unlike CTZ, no presynaptic effect of TCM has been reported (*40*). TCM increased the frequency and amplitude of mEPSCs in P16 and P30 neurons (Fig. 3, B to G). In the cumulative probability curve of the mEPSC amplitudes, we observed a right shift after TCM application at both ages (Fig. 3, B and C) with an increase in the average mEPSC amplitude (Fig. 3D). The effect size of the increase at both ages was similar (Fig. 3E). The increase was accompanied by an increased half-width of mEPSCs, indicating the effective inhibition of AMPAR desensitization (fig. S4, A and B) that was of similar effect size at both ages (fig. S4C). In contrast, while TCM increased the mEPSC frequency at both P16 and P30 (Fig. 3F), the effect size at P30 was approximately twofold larger than at P16 (Fig. 3G). Thus, at P30, a larger portion of transmission events was restored with TCM. Furthermore, the cumulative distribution shift after TCM application appeared uniform, which was consistent with a uniform increase in mEPSC events of all sizes.

The TCM solvent dimethyl sulfoxide (DMSO) had no significant effect on mEPSC parameters (fig. S4, D to H), except a small decrease in mEPSC amplitude as we tested with interleaved control experiments (fig. S4I). These results indicate that the desensitization blocker TCM restores transmission sites under basal synaptic transmission. Given the sparsity of spontaneous vesicle fusions, it indicates that transmission sites can reside in a long-lasting unresponsive state.

### Restoring transmission sites was distinct from blocking desensitization

**Fig. 4.**
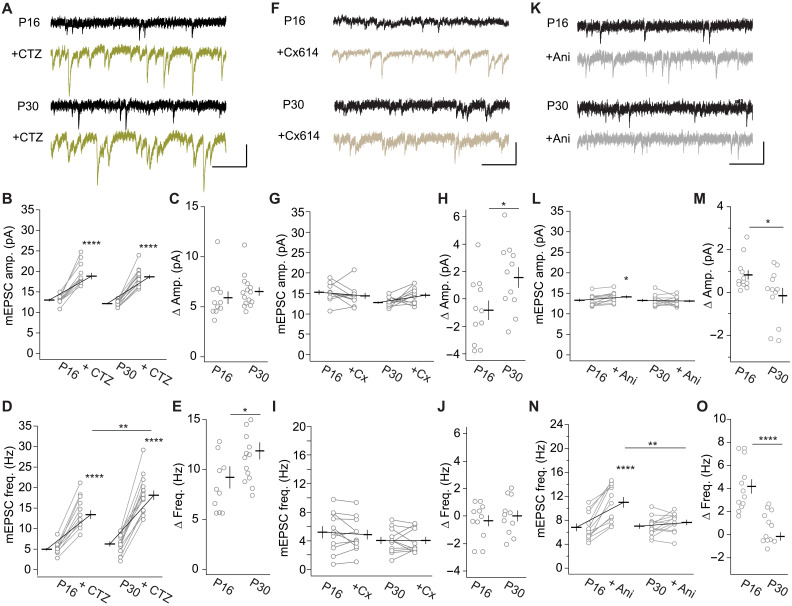
Distinct effects of desensitization inhibitors on desensitization and restoring transmission sites. (**A**) AMPAR-mediated mEPSC recorded at V1 L2/3 neurons from P16 and P30 mice before and after CTZ application. Summary graph of mEPSC amplitude (**B**), amplitude increase (**C**), frequency (**D**), and frequency increase (Δ*Freq*.) (**E**) with CTZ at the indicated ages. P16 (10/3) and P30 (15/4). (B) *F*_23,23_ = 2.296, *P* < 0.05; ACSF versus CTZ: *F*_1,23_ = 258.8, ****P < 0.0001; P16 versus P30: *F*_1,23_ = 0.818, *P* = 0.38; interaction: *F*_1,23_ = 0.837, *P* = 0.37. (C) *T*_23_ = 0.915, *P* = 0.37. (D) *F*_23,23_ = 3.180, *P* < 0.01; ACSF versus CTZ: *F*_1,23_ = 258.8, ****P < 0.0001; P16 versus P30: *F*_1,23_ = 7.148, *P* < 0.05; interaction: *F*_1,23_ = 7.414, *P < 0.05; P16 CTZ versus P30 CTZ: *P*_adj_ < 0.001. (E) *T*_23_ = 2.723, *P < 0.05. (**F**) AMPAR-mediated mEPSC recorded at V1 L2/3 neurons from P16 and P30 mice before and after Cx614 application. Summary graph of mEPSC amplitude (**G**), amplitude increase (**H**), frequency (**I**), and frequency increase (Δ*Freq*.) (**J**) with Cx614 at the indicated ages. P16 (11/3) and P30 (11/3). (G) *F*_20,20_ = 0.909, *P* = 0.58; ACSF versus Cx614: *F*_1,20_ = 4.194, *P* = 0.054; P16 versus P30: *F*_1,20_ = 0.564, *P* = 0.46; interaction: *F*_1,20_ = 4.30, *P* = 0.051. (H) *T*_20_ = 2.47, *P < 0.05. (I) *F*_20,20_ = 1.160, *P* = 0.37; ACSF versus Cx614: *F*_1,20_ = 3.122, *P* = 0.0925; P16 versus P30: *F*_1,20_ = 0.0747, *P* = 0.79; interaction: *F*_1,20_ = 0.0728; *P* = 0.79. (J) *T*_20_ = 0.601, *P* = 0.55. (**K**) AMPAR-mediated mEPSC recorded at V1 L2/3 neurons from P16 and P30 mice before and after aniracetam (Ani) application. Summary graph of mEPSC amplitude (**L**), amplitude increase (**M**), frequency (**N**), and frequency increase (Δ*Freq*.) (**O**) with aniracetam at the indicated ages. P16 (12/3) and P30 (12/4). (L) *F*_22,22_ = 4.559, *P* < 0.001; ACSF versus Ani: *F*_1,22_ = 2.576, *P* = 0.12; P16 versus P30: *F*_1,22_ = 1.413, *P* = 0.25; interaction: *F*_1,22_ = 5.327, *P < 0.05. (M) *T*_22_ = 2.308, *P < 0.05. (N) *F*_22,22_ = 5.006, *P* < 0.001; ACSF versus Ani: *F*_1,22_ = 42.98, ****P < 0.0001; P16 versus P30: *F*_1,22_ = 3.711, *P* = 0.067; interaction: *F*_1,22_ = 23.77, ****P < 0.0001; P16 aniracetam versus P30 aniracetam: **P_adj_ < 0.001. (O) *T*_22_ = 4.875, ****P < 0.0001. (A, F, and K) Scale bars, 200 ms and 20 pA.

A chronically desensitized state of AMPARs is not known, and the desensitized state is quickly recovered when glutamate is cleared (*41*). Furthermore, the ampakine Cx614 inhibits desensitization of AMPARs but does not affect mEPSC frequency in cultured neurons (*42*). Hence, to test whether the restoration of transmission sites was distinct from the common function of inhibiting desensitization, we used different desensitization inhibitors. CTZ is like TCM, a benzothiazide and a potent inhibitor of AMPAR desensitization. CTZ (100 μM) increased the frequency and amplitude of mEPSCs in P16 and P30 neurons (Fig. 4, A to E, and fig. S5, A and B). In the cumulative probability curve of the mEPSC amplitudes, we observed a right shift after CTZ application at both ages (fig. S5, A and B) with an increase in the mEPSC amplitude (Fig. 4B). The effect size of the increase at both ages was similar (Fig. 4C). The increase was accompanied by an increased half-width of mEPSCs, indicating the effective inhibition of AMPAR desensitization (fig. S5, C and D), which was of similar effect size at both ages (fig. S5E). In contrast, while CTZ increased mEPSC frequency at both P16 and P30 (Fig. 4D), the effect size at P30 was larger than at P16 (Fig. 4E). Thus, at P30, a larger portion of transmission events was restored with CTZ like with TCM, indicating that TCM and CTZ acted in a similar manner.

Cx614 is chemically different. It inhibits AMPAR desensitization similarly to CTZ but with less selectivity between flip and flop isoforms and, in addition, inhibits deactivation more than CTZ (*43*). Cx614 (30 μM) increased the half-width of mEPSCs at both P16 and P30 (fig. S5 H to J), consistent with the effect of Cx614 to inhibit desensitization and deactivation of AMPARs. The mEPSC amplitudes before and after Cx614 application at P16 were similar (Fig. 4, F and G), whereas at P30, the increase was bigger than at P16 (Fig. 4H). Nevertheless, mEPSC frequencies at either age before and after Cx614 application were similar (Fig. 4, I and J). Thus, while Cx614 was effective in inhibiting desensitization, comparable to TCM and CTZ, Cx614 did not restore additional synaptic transmission sites. If the unresponsive sites would have been due to desensitization, we would have expected a frequency increase with Cx614. Thus, the dissociation of the effect of Cx614 and TCM or CTZ on mEPSC frequency suggested that restoring transmission with TCM and CTZ was distinct from inhibiting desensitization.

To test whether the dissociation between desensitization inhibition and lack of effect on mEPSC frequency was specific for Cx614, we also compared the effects of aniracetam, a chemically different drug. Aniracetam also inhibits AMPAR desensitization and increases mEPSC amplitude but was not reported to affect mEPSC frequency (*44*, *45*). Aniracetam (2 mM) increased the half-width of mEPSCs similarly at P16 and P30 (fig. S5, M to O), consistent with aniracetam inhibiting desensitization of AMPARs. While aniracetam increased mEPSC amplitude at P16 by less than 1 pA, it did not at P30 (Fig. 4, L and M). Aniracetam increased mEPSC frequency at P16 almost twofold (Fig. 4, N and O). In contrast, at P30, the mEPSC frequency before and after aniracetam application was similar. As the half-width increase was similar at P16 and P30, a different amount of desensitization inhibition at the two ages could not account for the difference in aniracetam’s effect on mEPSC amplitude. The selective frequency increase by aniracetam at P16 was unlikely caused by the amplitude increase, because the amplitude increase by CTZ was five times bigger than by aniracetam, but the frequency increase was only twice bigger with CTZ compared to aniracetam. Cx614 increased mEPSC amplitude at P30 similarly to aniracetam did at P16. Nevertheless, we did not observe a frequency increase with Cx614 at P30. Furthermore, the mEPSC amplitude increase by CTZ and TCM was similar at P16 and P30, whereas the frequency increase was bigger at P30 than at P16. Thus, there was no correlation between the effects of the different desensitization inhibitors on mEPSC amplitude and frequency. The frequency increase was thus likely distinct from the effect of the drugs on desensitization. Notably, aniracetam was less potent in restoring transmission than CTZ and TCM and was ineffective at P30.

### The restoration of transmission sites spans over the entire amplitude range and was not dominated by the recovery of small mEPSCs above the detection threshold

As we used for the detection of mEPSCs a threshold of 8 pA, an amplitude increase might improve the detection and thus increase the apparent frequency. However, the different effect sizes of TCM and CTZ on the frequency at P16 versus P30 with a similar amplitude increase indicated that the amplitude increase could not be the only reason for the observed frequency increases and that specific unresponsive transmission sites additionally existed that can be pharmacologically restored. To quantify a potential contribution of amplitude increases to the increase in mEPSC frequency, we downscaled the mEPSC events under the TCM condition as those include events that may have been lifted above the threshold of mEPSC detection. To evaluate the contribution of thresholding, we analyzed a subgroup of our data in which the recording noise was low enough to reliably detect events with a threshold of 5 pA. Selecting the 13 cells from P16 and P30 with detectable events of 5 pA did not affect our central observations. TCM increased mEPSC amplitude at P16 and P30 in comparison to ACSF (fig. S6A). In contrast, the increase in frequency was bigger at P30 compared to that at P16 (fig. S6, B and C).

**Fig. 5.**
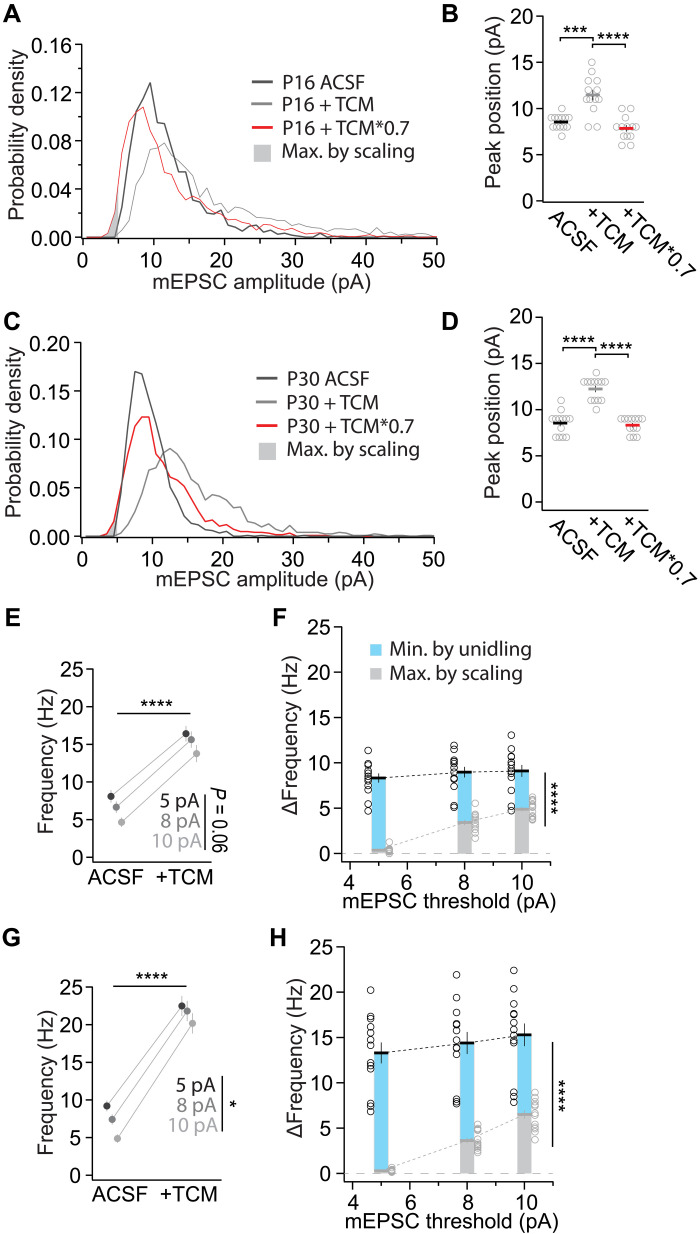
The frequency increase by TCM is minimally affected by amplitude scaling and detection threshold. (**A** and **C**) Sample probability density histograms of AMPAR-mediated mEPSCs at P16 (A) and P30 (C), recorded in ACSF, after TCM application and calculated events by multiplying the amplitude of TCM events by 0.7. The detection threshold was set to 5 pA and calculated events that are smaller than that are those that represent the maximum of events that could contribute to the frequency increase because of the upscaling of amplitudes by TCM. P16 (13/8) and P30 (13/7). (**B** and **D**) Summary graph of peak position of the probability density histograms at P16: (B) *F*_1.183,14.19_ = 48.70, *P* < 0.0001; ACSF versus TCM: ***P < 0.001; ACSF versus TCM*0.7: *P*_adj_ = 0.13; TCM versus TCM*0.7: ****P_adj_ < 0.0001; or P30: (D) *F*_1.831,21.97_ = 84.00, *P*_adj_ < 0.0001; ACSF versus TCM: ****P_adj_ < 0.0001; ACSF versus TCM*0.7: *P*_adj_ = 0.82; TCM versus TCM*0.7: ****P_adj_ < 0.0001. (**E** and **G**) Effect of the detection threshold on the frequency of events in ACSF or TCM at P16 (E): *F*_36,36_ = 9.052, *P* < 0.0001; treatment: *F*_1,36_ = 674.5, ****P < 0.0001; threshold: *F*_2,36_ = 3.043, *P* = 0.06; interaction: *F*_2,36_ = 0.507, *P* = 0.61; or P30: (G) *F*_36,36_ = 2.031, *P* < 0.05; treatment: *F*_1,36_ = 422.0, ****P < 0.0001; threshold: *F*_2,36_ = 3.801, *P* < 0.05; interaction: *F*_2,36_ = 0.686, *P* = 0.51. (**F** and **H**) Effect of the detection threshold on the potential maximum contribution of scaling to TCM-mediated frequency increase at P16 (F) or P30 (H). The upper values (open circles illustrating single neurons and bar illustrating the means ± SEM) depict the total frequency increase, while the gray symbols depict the frequency increase that could be contributed maximally by amplitude scaling. The remaining frequency increase (light blue) is due to a mechanism referred to as unidling. Two-factor ANOVA of threshold and frequency increase by either maximum potential frequency increase by scaling or total frequency increase. (F) Threshold: *F*_2,72_ = 18.57, ****P < 0.0001; Δ*Freq*. versus scaling; *F*_1,72_ = 263.4, ****P < 0.0001; interaction: *F*_2,72_ = 9.016, *P* < 0.001. (H) Threshold: *F*_2,72_ = 10.86, ****P < 0.0001; Δ*Freq*. versus scaling: *F*_1,72_ = 227.5, ****P < 0.0001; interaction: *F*_2,72_ = 2.856, *P* = 0.064.

We plotted the probability density histogram of the mEPSC amplitude distribution at P16 and P30 before and after TCM application (Fig. 5, A and C). In ACSF for both ages, the distribution was positively skewed with a peak that shifted to the right with TCM (Fig. 5, B and D). The shift of the peak to the right with a magnitude like the increase in average mEPSC amplitude after TCM application indicated that events across the amplitude range were restored. Otherwise, if primarily small events would have been lifted above the detection threshold, the distribution peak might have even been left shifted.

TCM increased the mEPSC amplitude by ~40%. To calculate which of the mEPSC events in TCM would have been below the detection threshold of 5 pA, we multiplied each event with the scalar of 0.7, the inverse of the 40% increase (Fig. 5, A and C). The peak of the downscaled distribution was at a similar value to that of the ACSF event distribution (Fig. 5, B and D). If the TCM events would have recovered primarily small events that then emerged from below the detection threshold, the peak of the downscaled distribution should have been smaller than that of the ACSF distribution. Thus, our result was consistent with no major contribution of amplitude scaling to the frequency increase by TCM. The fraction of events that shifted below the detection threshold was small (Fig. 5, A and C).

We primarily used an 8-pA threshold for mEPSC detection. Thus, some of the events were cut off from detection and might have had a bigger contribution to the frequency increase when the amplitude was increased after TCM application. To test how different detection thresholds affected mEPSC frequencies before and after TCM application, we determined the number of events with different thresholds in ACSF and TCM for P16 and P30. At both ages, the frequency increased after TCM application (Fig. 5, E and G). At P16, the reduction of mEPSC frequencies with increased threshold had a trend, whereas at P30, the reduction was significant. Thus, the different thresholding had a rather small effect on mEPSC frequencies. Notably, the mEPSC frequency increased by a similar magnitude between ACSF and TCM, as the lines were almost parallel and the interaction between treatment and threshold was not significantly affected, indicating that the increase in frequency was primarily due to events spread across all amplitudes and not by increased detection of primarily small events, which would have affected the slopes of the frequency increases with different thresholds.

We further quantified the potential contribution of amplitude scaling to the frequency increase. After multiplying their amplitude with the scalar of 0.7, we determined the number of events in TCM that fall below the corresponding detection threshold. The corresponding frequency was what scaling could maximally contribute to. The difference to the measured frequency was then due to another mechanism (Fig. 5, F and H). As described above, the total frequency increase after TCM application was independent of the detection threshold up to 10 pA. The maximal possible contribution of scaling to the frequency increase increased with the threshold increase. The maximal possible contribution to the frequency increase by scaling was smaller than the total frequency increase for all thresholds that we used (*P*_adj_ < 0.0001 for all comparisons at P16 or P30). Thus, while the TCM-related amplitude increases likely contributed to the frequency increase minimally, most of the increase was due to previously undetectable events that were restored by TCM. We refer to the transmission sites that are restored with TCM as idle-able in the following.

To summarize, both TCM and CTZ unidled more transmission sites at P30 than P16. As the control mEPSC frequency at P16 and P30 was similar and, only after the incubation with the desensitization blockers, at P30, the frequency was higher than at P16, our results were consistent with a developmental increase of transmission sites. However, during development, more of the miniature transmission sites were idled to maintain a constant frequency of mEPSCs that we refer to as synapse idling plasticity. This increase in idling occurred over the same developmental time course as the increase in silent synapse maturation.

### Two distinct populations of mEPSCs had different mean quantal amplitudes with the bigger one including idle-able transmission

**Fig. 6.**
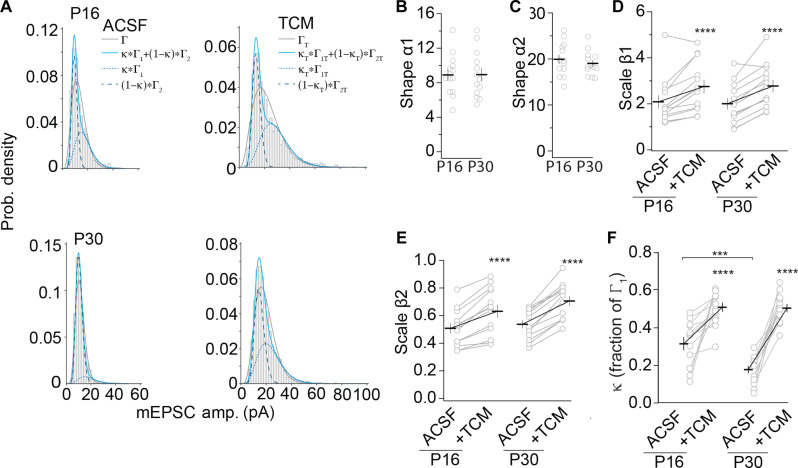
TCM treatment reveals distinct population of minisynaptic events. (**A**) Samples of single (black solid line) and mixed two (blue solid line) gamma fit of the mini-AMPAR EPSC amplitude probability density distribution (gray bars) in neurons from P16 (top) and P30 (bottom) animals in ACSF (left) and TCM (right). The two components of the gamma fit are depicted by dotted blue lines. (**B** and **C**) Summary graphs of the shape parameters α1 (B) and α2 (C) of the gamma fit in ACSF. (B) *T*_24_ = 0.032, *P* = 0.985. (C) *T*_24_ = 0.71, *P* = 0.48. (**D** and **E**) Summary graphs of the scale parameters β1 (D) and β2 (E) of the gamma fit in the depicted conditions. (D) *F*_24,24_ = 14.62, *P* < 0.0001; age: *F*_1,24_ = 0.000348, *P* = 0.985; treatment: *F*_1,24_ = 55.01, ****P < 0.0001; interaction: *F*_1,24_ = 0.282, *P* = 0.60. (E) *F*_24,24_ = 22.86, *P* < 0.0001; age: *F*_1,24_ = 1.023, *P* = 0.32; treatment: *F*_1,24_ = 179.1, ****P < 0.0001; interaction: *F*_1,24_ = 4.34, *P* < 0.05. (**F**) Summary graph of the weight (κ) of first component of the mixed gamma fit in the indicated experimental groups. *F*_24,24_ = 0.647, *P* = 0.85; age: *F*_1,24_ = 9.1, **P < 0.01; treatment: *F*_1,24_ = 82.94, ****P < 0.0001; interaction: *F*_1,24_ = 5.34, *P* < 0.05, ACSF P16 versus ACSF P30: ***P_adj_ < 0.001; TCM P16 versus TCM P30: *P*_adj_ = 0.93. Dots represent values of single neurons with the line representing the means ± SEM. P16 (13/8) and P30 (13/7).

Evoked EPSCs and mEPSCs have been reported to originate from different synapses and/or activate distinct NMDA receptors (*21*, *23*, *27*). Whether mEPSCs themselves are heterogeneous rather than one uniform distribution of quantal sizes is unknown. In contrast to the normal distribution of mEPSC amplitudes at the neuromuscular junction, the distribution is positively skewed at central synapses (*46*, *47*). This distribution originates from the variability of mEPSC of single synapses, and the distribution can be better described by a gamma (Γ) distribution (*5*, *7*, *46*, *48*). We fitted the probability density histograms of the amplitude distribution with a gamma function for P16 and P30 (Fig. 6A). The parameters α and β describe the shape and scale of the distribution, respectively, with α times β equaling the mean. With α > 1, the distribution is positively skewed, while β determines how the distribution is stretched rightward. We found that a weighted two gamma distribution (blue line) resulted in a better fit compared to a single gamma fit (gray line) for all distributions of single neurons (Fig. 6A and table S1), capturing both the initial peak and the shoulder that was more pronounced in some distributions. This result indicated two types of mEPSC distributions, which was consistent with two distinct transmission sites.

The shapes of the gamma distribution, α1 and α2, did not differ between P16 and P30 in ACSF (Fig. 6, B and C). The scale factors of the gamma distribution, β1 and β2, also did not differ between P16 and P30 in ACSF (Fig. 6, D and E). Thus, during development, the distribution of the two types of mEPSCs did not change.

We then tested whether the change in the scale factor and, potentially, the weight of the two populations can capture the shift in the distribution of the amplitudes in TCM compared to those in ACSF from the same cell. With the shape factors constrained with the fitting parameters from ACSF recordings, scale factors and weights were obtained from fitting the TCM recordings from the same cell. The fitted scale factors increased for the TCM treatment in comparison to ACSF and were alike for β1 at P16 and P30 (Fig. 6, D and E). For β2, we observed an interaction between treatment and age, but β2 was not significantly different between ages (ACSF: P16 versus P30, *P*_adj_ = 0.58; TCM: P16 versus P30, *P*_adj_ = 0.16). This result was consistent with the TCM effect on AMPAR channel properties enhancing AMPAR EPSC amplitudes by ~30 to 40% and with no difference at P16 versus P30.

The fitted weight factor κ of the population captured by Γ_1_ was significantly higher at P16 compared to that at P30 (Fig. 6F). Moreover, κ increased after TCM application in comparison to ACSF at both P16 and P30 but to a similar value. These results were consistent with two distinct populations of mEPSCs of which the population captured by Γ_1_ included the idle-able transmission sites, whose weight was increased after TCM application and more so at P30, as more sites were idled (Fig. 3). The first population had a bigger mean amplitude of 16.9 ± 1.1 pA at P16 or 16.1 ± 0.7 pA at P30 in comparison to the second population with a mean amplitude of 9.69 ± 0.22 pA at P16 or 9.98 ± 0.26 pA at P30 (P16: *T*_24_ = 6.69, *P* < 0.001; P30: *T*_24_ = 8.11, *P* < 0.001). Thus, mEPSCs in V1 L2/3 pyramidal neurons were composed of two distinct populations with different mean quantal sizes and distributions of which the one with higher amplitude contained idle-able transmission sites. Notably, during development, the fraction of Γ_1_ that contributes to transmission reduced from P16 to P30, but after TCM application, it was similar. This property was consistent with idling contributing to the homeostasis of mEPSC frequency.

### Effects of TCM on miniature synaptic transmission versus evoked synaptic transmission differed

**Fig. 7.**
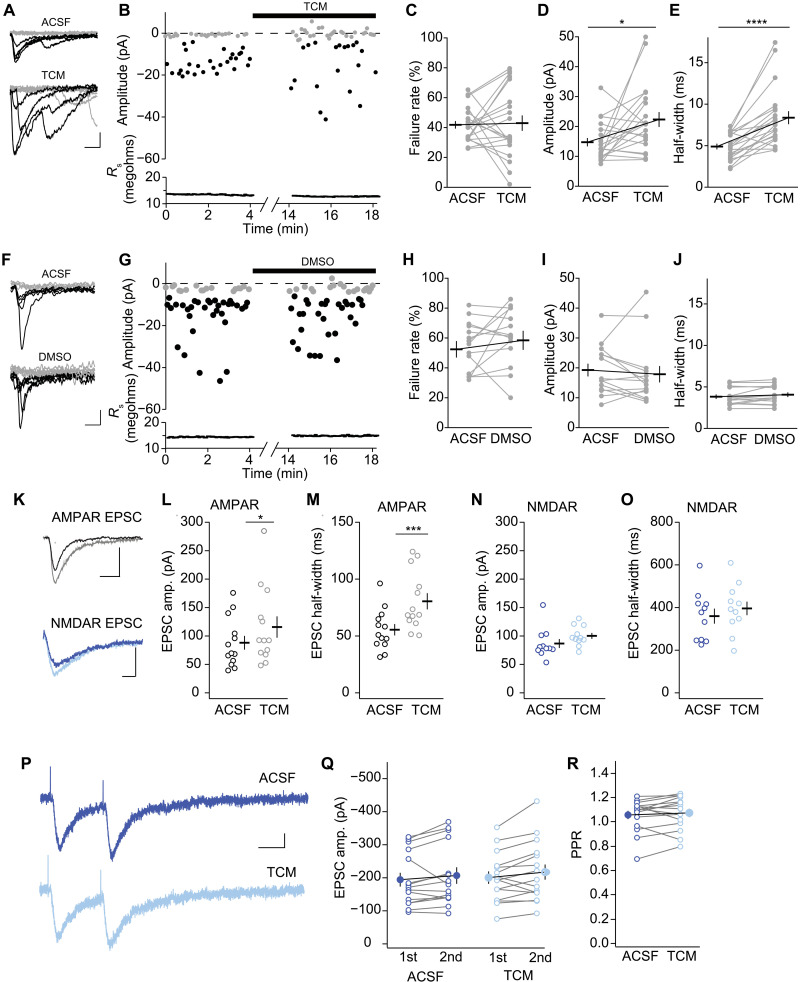
TCM affected the time course of evoked AMPAR EPSCs but not quantal content. (**A** and **F**) Minimally evoked AMPAR-mediated EPSCs before and after TCM application (A) or before and after DMSO application (F) with failures or successes of transmission in gray or black, respectively. *R*_s_ is depicted at the bottom. Scale bar, 10 ms and 10 pA. Effect of TCM on failure rate (**B** and **H**), amplitude (**C** and **I**), and half-width (**D** and **J**). Values of individual cells are depicted as circles and average as a bar with SEM. (C) 20/5; *T*_19_ = 0.106, *P* = 0.92; (D) *T*_19_ = 2.594, *P < 0.05; (E) *T*_19_ = 5.440, ****P < 0.0001. (H) 14/6; *T*_13_ = 1.252, *P* = 0.23; (I) *T*_13_ = 0.752, *P* = 0.47; (J) *T*_13_ = 2.004, *P* = 0.07. (**K** to **O**) Sample traces and summary of amplitude and half-width of AMPAR and NMDAR EPSCs in neurons treated with TCM. (K) AMPAR-mediated EPSCs were recorded at *V*_*h*_ = −60 mV, and NMDAR-mediated EPSCs were recorded at *V*_*h*_ = −40 mV in the presence of NBQX (10 μM). Scale bar, 20 ms and 100 pA. Summary graphs of EPSC amplitude for AMPARs (L) and NMDARs (N) and EPSC half-width for AMPARs (M) and NMDARs (O). Means ± SEM are depicted together with values of individual cells as circles. (L) 13/3; *T*_12_ = 2.609, *P < 0.05; (M) *T*_12_ = 4.748, ***P < 0.001; (N) 11/3; *T*_10_ = 1.503, *P* = 0.16; (O) *T*_10_ = 2.118, *P* = 0.06. (**P**) Example traces of NMDAR-mediated EPSCs with paired-pulse stimulation (100-ms interstimulus) were recorded at *V*_*h*_ = −40 mV in the presence of NBQX in ACSF (top) and after TCM application (bottom). Scale bar, 50 ms and 50 pA. (**Q**) Summary graph of EPSC amplitude for first (1st) and second (2nd) stimuli in ACSF and after TCM application (last 25 sweeps were averaged). 15/3; *F*_28,28_ = 13.85, *P* < 0.0001; treatment: *F*_1,28_ = 1.299, *P* = 0.26. (**R**) Summary graph of PPR in ACSF and after TCM application. *T*_14_ = 1.211, *P* = 0.25.

We next tested whether the TCM effect was selective for mEPSCs or influenced also quantal content for evoked synaptic transmission. We stimulated L4-to-L2/3 synaptic inputs onto L2/3 pyramidal neurons of P14 to P20 mice with minimal stimulation. After 50 sweeps in ACSF, we applied 500 μM TCM and, after 10 min, recorded another 50 sweeps (Fig. 7, A and B). The failure rate before and after TCM application was alike (Fig. 7C). As the failure rate did not change, TCM unlikely influenced release probability, nor did it recover new transmission sites of evoked transmission. Both potency (the mean amplitude of successes) and half-width of the EPSCs increased after TCM application (Fig. 7, D and E) to a similar degree, as measured for mEPSCs (Fig. 3 and fig. S4), consistent with TCM inhibiting AMPAR desensitization. Also, for the solvent DMSO, the failure rate, potency, and half-width before and after application were similar (Fig. 7, F to J). The contrast of the lack of TCM effect on responses to evoked quantal content and the robust effect of TCM on mEPSC frequencies suggested two distinct underlying transmission sites, which were differentially recruited for spontaneous transmission and evoked transmission.

To test whether the TCM effect was selective for AMPAR-mediated synaptic transmission, we stimulated axons in L4 to evoke both AMPAR and NMDAR EPSCs. For AMPAR EPSCs, we clamped L2/3 pyramidal neurons at *V*_*h*_ = −60 mV when NMDARs are blocked by Mg^2+^ (Fig. 7K). After 20 to 30 sweeps in ACSF, we applied TCM and recorded a similar number of EPSCs. The amplitude and half-width of AMPAR EPSCs increased after TCM application (Fig. 7, L and M), consistent with the result of minimal stimulation (Fig. 7A). In a different set of neurons, we recorded NMDAR EPSCs at *V*_*h*_ = −40 mV and blocked AMPARs with 10 μM NBQX (Fig. 7K). Neither the amplitude nor the half-width of NMDAR EPSCs was affected significantly by TCM (Fig. 7, N and O). These results indicated a similar effect of TCM on evoked AMPAR-mediated synaptic transmission to the amplitude increase for mEPSC. However, no change was detected in magnitude of the size, which we observed for the frequency increase in mEPSC, further supporting the additional effect on mEPSCs for unmasking transmission sites that was not observed for evoked EPSCs. NMDAR EPSCs were not affected, which excluded a major effect of TCM on evoked presynaptic release in the L4-to-L2/3 pyramidal neural pathway.

### TCM did not affect presynaptic release probability

CTZ was proposed to increase the release probability of evoked synaptic transmission at least at some synapses (*3*, *37*–*39*). The lack of TCM effects on NMDAR EPSCs and failure rates suggested that it was not the case for L2/3 visual cortical pyramidal neurons under our experimental conditions. Nevertheless, a developmental decrease in release probability could equilibrate at least partially the increase in the number of unsilenced transmission sites. Release probability is the likelihood that a presynaptic action potential triggers synaptic vesicle fusion (*20*). We assessed whether the release probability of AMPAR EPSCs changed from P10 over P14 to P30. We measured the ratio of amplitudes between two consecutively evoked EPSCs with an interstimulus interval of either 50 or 100 ms, referred to as the paired-pulse ratio (PPR) (fig. S7A). The PPR of the second EPSC over the first EPSC was similar at all three developmental time points that we tested (fig. S7B), suggesting no change in release probability for AMPAR EPSCs.

We then tested the effect of TCM on *P*_r_ by two approaches on NMDAR EPSCs. These procedures have the advantage that TCM does not affect the NMDAR EPSC waveform. We measured NMDAR EPSC PPRs (Fig. 7P). Like the one-stimulus evoked EPSCs, the amplitude of NMDAR EPSCs was similar before and after TCM application (Fig. 7Q). The PPR was also similar before and after TCM application (Fig. 7R). We then tested *P*_r_ by the use-dependent blocking rate of the NMDAR open channel blocker MK-801 (*49*). NMDAR EPSCs progressively declined in the presence of 5 μM MK-801 with at least one time constant for high-*P*_r_ synapses and one time constant for low-*P*_r_ synapses (fig. S7C) (*49*). We fitted each time course by a double-exponential decay equation and determined two time constants and the amplitude of the fast component. All three parameters were similar between the ACSF group and the TCM group (fig. S7, D and E). Thus, in L2/3 pyramidal neurons, TCM did not have a measurable effect on release probability.

### TCM increased mEPSC frequency through postsynaptic mechanisms

Changes in mEPSC frequency have been commonly associated with presynaptic changes of fusogenicity (*2*, *50*). We observed an increase in mEPSC frequency of more than twofold after TCM application but no effect on *P*_r_ (Figs. 3 and 7 and fig. S7). Silent synapse unsilencing at the beginning of the critical period when the % of silent synapses is high is one postsynaptic mechanism to increase mEPSC frequency by increasing the postsynaptic reception of spontaneously fusing synaptic vesicles (*34*, *35*). CTZ was reported to increase mEPSC frequency in a similar range to what we observed. It has been explained by effects on *P*_r_ or on the improved detection of mEPSCs because of the increase in mEPSC amplitude by CTZ (*3*, *51*). However, both mechanisms did not account for the full effect of TCM on mEPSC frequency in L2/3 pyramidal neurons (Fig. 5).

To test whether TCM acts on spontaneous transmission selectively, we examined NMDAR mEPSCs as TCM does not influence the NMDAR function (Fig. 7 and fig. S7). Focusing on NMDAR mEPSC could thus enable us to distinguish between fusogenicity, a presynaptic effect, and AMPAR unidling, a postsynaptic effect. The time course of NMDAR mEPSCs is slower and reduces the accuracy of mEPSC detection compared to mEPSCs of AMPARs. Therefore, we used another method to estimate the changes of mEPSCs after drug treatments. We fitted a baseline for the current trace and measured the charge under the baseline (*52*). Treatment with TCM increased the AMPAR-mediated charge threefold in comparison to its charge in ACSF (fig. S8, A and B). Thus, using the charge instead of single-event detection as a measure resulted in a similar increase in AMPAR mEPSCs by TCM. Notably, in contrast to the single-event detection, this increase was affected by both the increase in quantal size and mEPSC frequency. As TCM had a bigger effect on mEPSC frequency than amplitude (Fig. 3), the increase in charge was dominated by the frequency increase, allowing us to use the effect on charge as an approximation for assessing the effects of TCM on AMPAR mEPSC frequency.

**Fig. 8.**
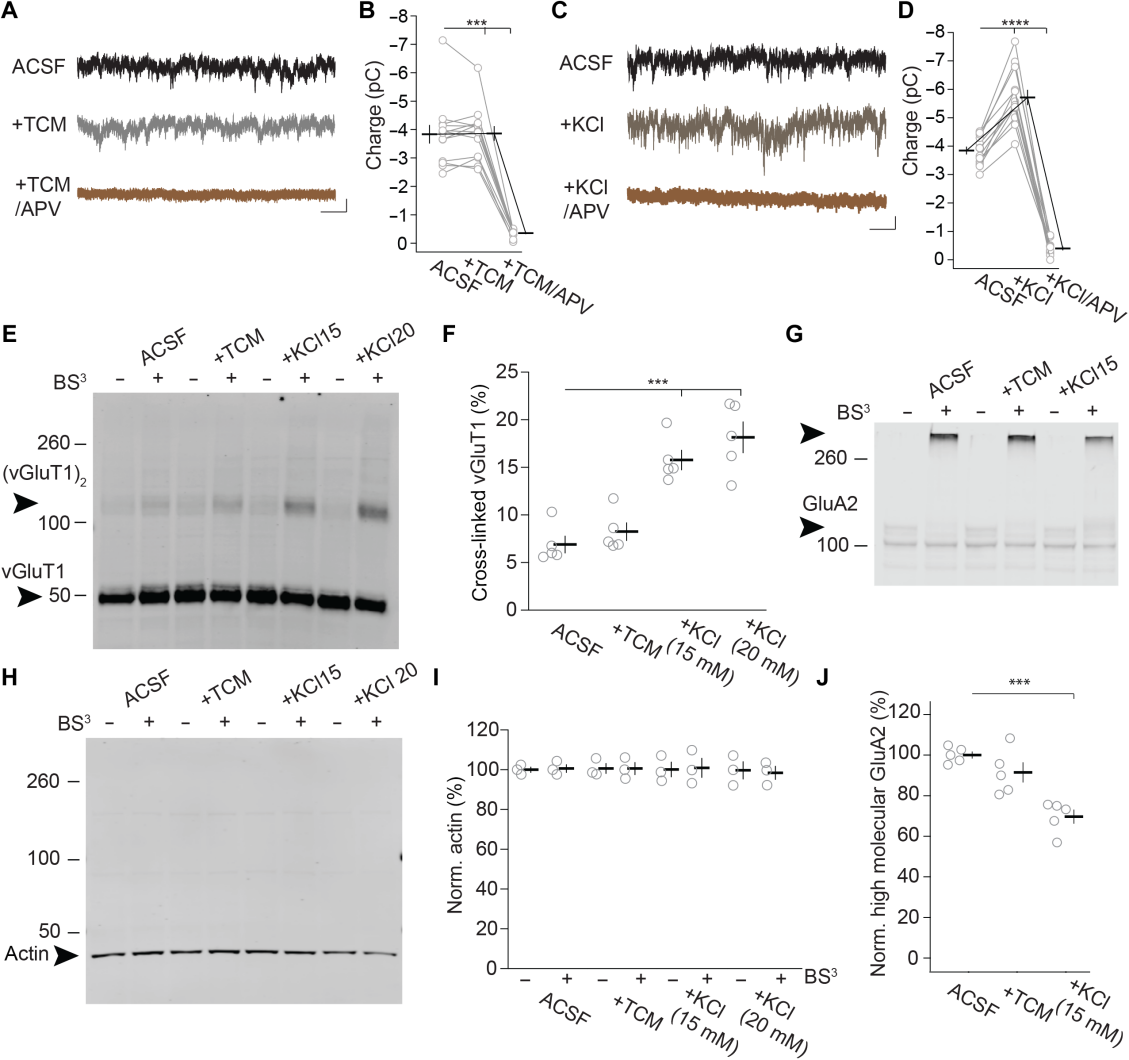
TCM selectively increases AMPAR-mediated mEPSC frequency. (**A** to **D**) NMDA mEPSCs were recorded at *V*_h_ = −40 mV and with NBQX and then with addition of TCM and followed by addition of 50 μM APV [(A) and (B)] or with addition of 15 mM KCl and followed by addition of APV [(C) and (D)]. Sample traces [(A) and (C)] and summary graph of charge transfer at the indicated conditions [(B) and (D)]. (B) 13/3; *F*_2,24_ = 148.3, *P* < 0.0001; ACSF versus TCM: *P*_adj_ > 0.99; TCM versus APV: ***P_adj_ < 0.01. (C) 13/4; *F*_2,24_ = 343.2, *P* < 0.0001; ACSF versus KCl: ****P_adj_ < 0.0001. Scale bar, 1 s and 10 pA. (**E**, **G**, and **O**) Immunoblot of visual cortex slices after treatment with the indicated conditions. (E) The monomer and putative dimer bands of vGluT1 were marked with arrowheads and vGluT1 or (vGluT1)_2_, respectively. (G) Actin was marked with an arrowhead. (**I**) The monomer and cross-linked bands of GluA2 were marked with arrowheads. Groups were treated with ACSF only for control, 500 μM TCM, 15 mM KCl, or 20 mM KCl in either the presence or absence of BS^3^. (**F**) The summary graph of (vGluT1)_2_ band intensity relative to the total (monomer plus dimer) band intensity was calculated as the percentage with values for single mice as circles and the means as a bar with SEM. 5/5; *F*_3,16_ = 22.92, *P* < 0.0001; ACSF versus TCM: *P*_adj_ = 0.75; ACSF versus 15 mM KCl: ***P_adj_ < 0.001; ACSF versus 20 mM KCl: ****P_adj_ < 0.0001. (**H**) Summary graph of pixel intensities of the immunoblot, decorated with an antibody against actin. For each condition, the pixel intensities of the monomer band were quantified. The values were normalized to the average of the ACSF without BS^3^ group. 5/5; *F*_3,16_ = 1.801, *P* = 0.16. (**J**) Summary graph of pixel intensities of the cross-linked GluA2 immunoreactive band. The cross-linked GluA2 band was quantified and normalized to the average of the ACSF with BS^3^ group. 5/5; *F*_2,12)_ = 18.34, *P* < 0.001; ACSF versus TCM: *P*_adj_ = 0.213; ACSF versus 15 mM KCl: ***P_adj_ < 0.001.

To isolate NMDAR mEPSCs, we performed recordings while blocking AMPARs with NBQX and with *V*_h_ = −40 mV to reduce Mg^2+^ binding to the NMDAR pore. In contrast to AMPAR mEPSCs, the charge of NMDAR mEPSCs after treatment with TCM was like that in ACSF (Fig. 8, A and B). We then added 50 μM d,l-2-amino-5-phosphonovaleric acid (APV) to estimate the charge that was mediated by the NMDARs. The residual charge was ~10%, indicating that ~90% of the charge was mediated by NMDARs. Thus, TCM had no significant effect on NMDAR mEPSCs. To confirm the sensitivity of our approach, we increased the fusogenicity by adding 15 mM KCl into ACSF. This treatment increased the charge mediated by NMDARs by ~50% (Fig. 8, C and D). APV (50 μM) removed most of the charge in the presence of KCl, confirming NMDARs as the mediators of the currents. Thus, while NMDARs could detect increases in fusogenicity, TCM had no effect on NMDAR-mediated mEPSCs, whereas it increased AMPAR-mediated mEPSCs, indicating a selective effect of TCM on AMPAR-mediated mEPSCs.

To further evaluate the effect of KCl on fusogenicity, we directly measured AMPAR mEPSC frequency before and after addition of 15 mM or 20 mM KCl (fig. S8, C and D). Both concentrations of KCl increased mEPSC frequency between three- and fourfold with 20 mM KCl having a bigger effect (fig. S8E). Thus, these concentrations of KCl had a similar effect on mEPSC frequency to TCM, indicating that our approach should have revealed an effect of TCM on fusogenicity if it was present.

As the effects of TCM so far relied on postsynaptic detection, we next targeted presynaptic terminals to estimate synaptic vesicle fusogenicity with a biochemical approach. We used a membrane-impermeable cross-linker that reacts with extracellular proteins. The electrophoretic mobility was changed through the cross-linking with other proteins, which allowed the estimation of the surface proteins after detection with specific antibodies (fig. S8F) (*53*). vGluT1, a biomarker of glutamate synaptic vesicles of pyramidal neurons (*54*), resides on synaptic vesicles of presynaptic terminals and is not accessible to the cross-linker BS^3^. However, if synaptic vesicles fuse, the synaptic vesicle membrane becomes part of the plasma membrane and vGluT1 can react with the cross-linker. The monomeric band of vGluT1 was prominent and did not reduce with cross-linking (Fig. 8E). A putative vGluT1 dimer, (vGluT1)_2_, was detectable at ~110 kDa, and the band intensity increased after cross-linking, indicating that it represented surface-exposed vGluT1 during the reaction time of 30 min. Some faint, higher-molecular-weight complexes were also detectable in the BS^3^ samples. The (vGluT1)_2_ band intensity was less than 20% of that of the monomer. Thus, only a small fraction of vGluT1 reached the surface to be cross-linked and had no detectable effect on the change of total vGluT1. The putative cross-linked dimer was used as a proxy of synaptic vesicle exocytosis. The fraction of (vGluT1)_2_ to the total (sum of monomer and putative dimer) intensity did not increase after TCM treatment but increased with 15 or 20 mM KCl, consistent with KCl increasing synaptic vesicle fusion events (Fig. 8F). Thus, TCM did not increase fusogenicity as measured by vGluT1 cross-linking.

As a control for extracellular cross-linking, we tested actin, an intracellular protein. We detected the monomeric actin by Western blotting (Fig. 8G). With or without BS^3^, we did not observe higher-molecular-weight bands. When quantifying the pixel intensities for the range of 50 to 260 kDa and normalizing to the mean value in the ACSF group without BS^3^, we did not observe any significant change among the groups (Fig. 8H), indicating that the cross-linker did not reach the inside of the neurons and the increase in vGluT1 cross-linking was due to an increase in synaptic vesicle fusion. Together, these results indicated that the BS^3^ cross-linking procedure allowed the detection of increased fusogenicity by KCl that caused a similar increase in mEPSC frequency to TCM, but TCM itself had no detectable effect on fusogenicity. Thus, the TCM-mediated increase in mEPSC frequency was primarily mediated by the postsynaptic recovery of idled transmission sites.

With the BS^3^ procedure, we also tested whether the number of AMPARs on the surface was influenced by the pharmacological procedures. We assessed the surface levels of GluA2, the AMPAR subunit that is most prevalent in the principal neuron synapses (*55*). Without BS^3^ treatment, the monomeric band of GluA2 had the expected molecular weight of 110 kDa (Fig. 8I). With BS^3^ treatment, the band intensity of the monomer decreased and a high-molecular-weight band immunoreactive for the GluA2 antibody appeared, indicating that most of GluA2 was expressed on the surface and accessible to the cross-linker. The band intensity of the cross-linked GluA2 after TCM application was like that of the ACSF group (Fig. 8J). However, after 15 mM KCl, the cross-linked GluA2 band intensity was smaller than that for the ACSF group, indicating that the KCl treatment that caused increased glutamate release also affected GluA2 surface levels by reducing them. Together, these results reveal that TCM did not affect the surface levels of GluA2, indicating that there was no fast TCM-mediated increase in surface GluA2 levels, which could cause the mEPSC frequency increase.

Our results so far revealed that TCM acted postsynaptically to recover idled AMPARs in idle-able transmission sites that contributed to miniature synaptic transmission. In contrast, the other types of transmission sites were sampled with evoked transmission that did not respond to TCM, except for the change in AMPAR EPSC kinetics. These sites included the silent states, which were unsilenced along the critical period. Thus, we referred to these transmission sites as silenceable transmission sites. Together, our results indicated that two distinct types of transmission sites existed.

### Distinct parallel transmission sites existed on single spines

**Fig. 9.**
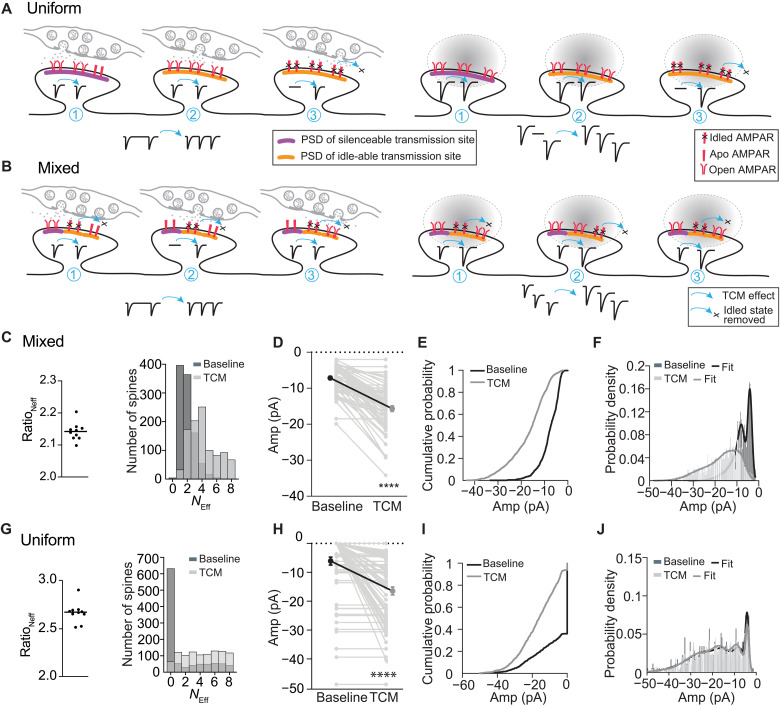
Simulation of two possible synapse configurations for silenceable and idle-able transmission sites. (**A**) Uniform and (**B**) mixed models of transmission sites on spines with presynaptic vesicle release (left) or with two-photon uncaging of caged glutamate onto the spine head (right). Idle-able transmission sites are depicted with an orange PSD, while silenceable transmission sites are depicted with a purple PSD. (A) Three synaptic states are depicted for the uniform configuration: unsilenced silenceable (1), unidled idle-able (2), and idled idle-able (3). Vesicle fusions can occur at any of the three positions of the active zone, but for simplicity, only one for each synaptic state is depicted. Glutamate (gray) uncaging is depicted as the oval on the right. AMPAR states are depicted in the legend in (A) and the TCM effect in the legend in (B). Synaptic responses of the individual spines are depicted as the inset in the spine and the change after TCM application (blue arrow) with the response on the right. [(A) and (B)] The synaptic responses as recorded for mEPSCs (left) or glutamate uncaging (right) before (left of arrow) and after TCM application (right of arrow) are depicted at the bottom. (**C** to **F**) Mixed model simulation. (**G** to **J**) Uniform model simulation. [(C) and (G)] Ratio of numbers of effective transmission sites (*N*_eff_) after TCM application to that of the baseline condition (left) and the histogram of number of spines out of total 1000 spine heads with different numbers of effective transmission sites at the baseline (dark gray) and with TCM application (light gray) (right). [(D) and (H)] Summary graphs: paired *t* test: ****P < 0.0001. [(E) and (I)] Cumulative probability and [(F) and (J)] probability density plots of EPSC amplitudes of simulated single spine heads under the baseline condition (black) and after TCM application (gray). Lines in (F) and (J) indicate the mixed Gaussian fit of the EPSC amplitude distribution.

After identifying two distinct types of transmission sites, the question arose whether they were at the same spine/synapse or on separate spines/synapses. Synaptic glutamate in the neuropil spreads beyond synapses, and spillover was observed to activate AMPARs at neighboring synapses (*56*, *57*). However, glutamate spread at other synapses is more confined to activate only a fraction of AMPARs in a synapse (*58*). Thus, it remained unknown whether glutamate from a single vesicle can reach all AMPARs of a synapse or only a subfraction, thus whether idle-able and silenceable transmission sites coexisted (mixed) at single synapses or whether they constituted separate uniform synapses (Fig. 9, A and B). To account for the effect of TCM on mEPSC frequency, the following constraints on the synaptic configuration would be required. If different transmission sites localized on separate spines, then we would have all idled, unidled, or silenceable sites (Fig. 9A). In such a configuration, TCM would recover the idled transmission sites to increase mEPSC frequency and the effect of TCM on mEPSC amplitude would be small as TCM would render nonresponsive, idled AMPARs fully responsive. Alternatively, if idle-able and silenceable transmission sites are mixed on the same spine and some were idled, then TCM would recover those and increase mEPSC frequency (Fig. 9B). We observed preferentially an increase in mEPSC frequency and not amplitude despite the modest increase mediated by inhibiting AMPAR desensitization. This observation implies the following features for the mixed transmission sites: They need to be distinct with their own AMPAR clusters and without major cross-talk.

These two scenarios could be differentiated if glutamate is applied to single spines to reach all AMPARs (Fig. 9, A and B, right). In the uniform model with idle-able and silenceable transmission sites on different spines, after TCM application, spines with previously idled AMPARs would become responsive and increase the success rate of responses. TCM would have little effect on the response size of previously responsive spines (Fig. 9A, right). In contrast, in the mixed model, the size of the response amplitude would increase after TCM application (Fig. 9B, right). The success rate of responses would not be largely affected as each spine would contain responsive AMPAR clusters despite being in a silent synapse.

To quantitatively distinguish between these possibilities, we simulated a mixed model and a uniform model in silico (for the simulation scheme, see fig. S9). In the mixed model, we assumed that each spine head was occupied by one to two silenceable transmission sites, with each site having a 20% chance of a silent state and 80% chance of an unsilenced state, based on our observation of 20% of silent synapses at P30 (Fig. 1) (*11*, *12*, *15*). With each silenceable site, one to three idle-able sites were assigned to the same spine head, and the chance of being idled increased if an unsilenced site existed on the spine head based on the correlation between silent synapse maturation/unsilencing and idling of idle-able transmission sites (Figs. 1 to 3). In the uniform model, each spine head was occupied by one to eight transmission sites, with the restriction that the mode and state of all transmission sites on each single spine were identical. The chance of assignment was 5% silent silenceable, 20% unsilenced silenceable, 60% idled idle-able, and 15% unidled idle-able sites to account for the % of silent synapses and the observed increase in mEPSC frequency by TCM application (Fig. 3). One thousand spine heads were simulated in each iteration, and 10 iterations were simulated for the analysis. In both models, TCM application increased the number of effective transmission sites by 2.2- to 2.5-fold (Fig. 9, C and G), consistent with the effect of TCM on mEPSC frequency (Fig. 3F), which depends on the number of transmission sites. In the mixed model, the number of spines that contained more effective transmission sites increased after TCM application with a right shift of the distribution of the number of effective transmission sites (*N*_Eff_) (Fig. 9C). In contrast, in the uniform model, the overall distribution pattern did not change, but the number of spines that contained zero effective transmission sites (*N*_Eff_ = 0) decreased after TCM application (Fig. 9G). To illustrate the effect of TCM application, we randomly selected 70 spines from one simulation and plotted the response of the spine upon brief glutamate application (mimicking the scenario with two-photon uncaging of glutamate), under the baseline condition, and after TCM application. In both models, TCM application induced an increase in the response amplitude by about twofold (Fig. 9, D and H). However, the pattern of response size increases differed between the two models, as predicted from the model scheme (Fig. 9, A and B). TCM increased the spine response regardless of the initial response size in the mixed model, whereas it only had effects on some spines with no initial responses in the uniform model (Fig. 9, D and H). This is also illustrated in the *N*_Eff_ distribution plot (Fig. 9, C and G) and the cumulative probability of spine response amplitudes (Fig. 9, E and I). In the uniform model, about 60% of spines exhibited no response at the baseline, and the proportion of nonresponsive spines decreased to about 5% after TCM application. We filtered out the nonresponders and illustrated the probability density of the spine amplitude responses in the mixed model and the uniform model (Fig. 9, F and J). We fitted the distribution with a multi-Gaussian function on the basis of an independent, multitransmission site configuration at synapses. The simulation predicted that in the mixed model, the distribution of spine amplitude responses shifted to bigger amplitudes, broadening the probability density distribution. The proportion of smaller responses decreased, whereas the proportion with bigger amplitudes increased (Fig. 9F). In the uniform model, the distribution patterns of amplitude responses were not different between the baseline and after TCM application (Fig. 9J), and only the proportion of responding spines increased (Fig. 9G). The simulation of the two models provided the prediction to distinguish between the synapse configurations (mixed versus uniform) for the experimental outcome of activating AMPARs on single spines.

**Fig. 10.**
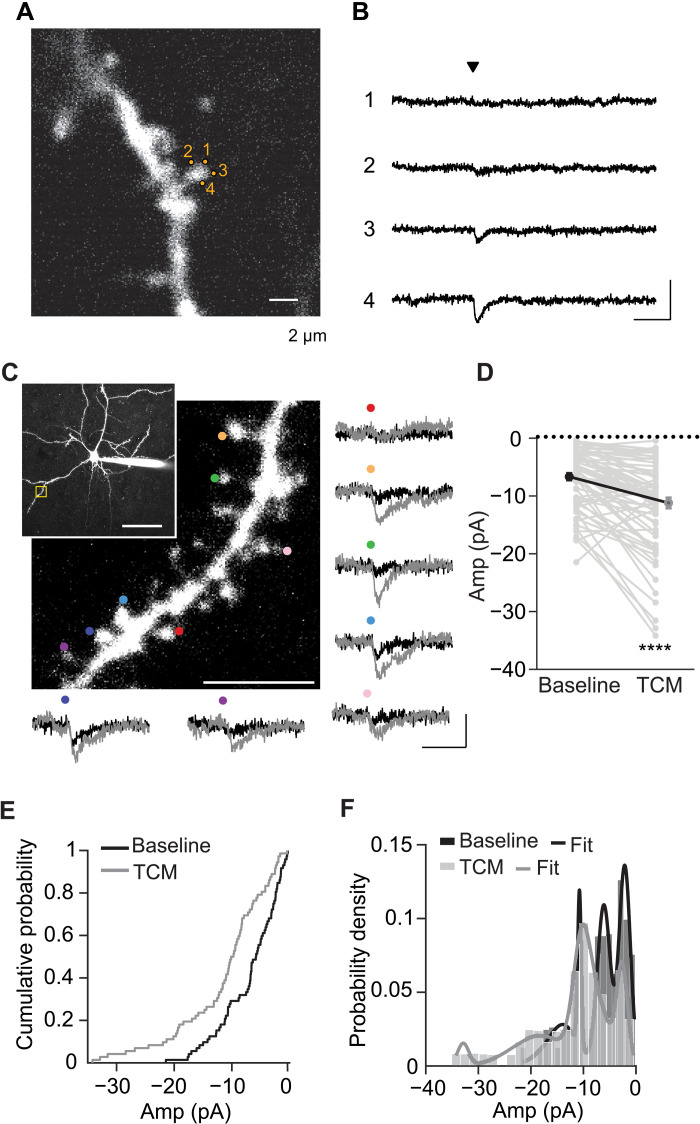
Mixed transmission sites exist on single spines. (**A**) Two-photon image of a basal dendrite of L2/3 neuron loaded with Alexa 594. In this example, the spine is ~40 μm below the surface of the slice. Scale bar, 2 μm. (**B**) Voltage-clamp traces corresponding to single uncaging trials recorded sequentially at the locations indicated by the orange spots in (A). A 0.5-ms uncaging pulse was delivered at the time indicated by the arrowhead. Laser intensity was the same for each trial. Scale bar, 50 pA and 20 ms. (**C**) Sample image of L2/3 pyramidal neuron (inset) and enlarged dendritic segment (yellow box). Representative sample traces (color coded to match the spine with uEPSC) of some spines are depicted with uEPSC before TCM (black) and after TCM application (gray). Scale bar, 10 pA and 20 ms; 5 and 50 μm (inset). (**D**) Summary graph, (**E**) cumulative probability, and (**F**) probability density plots of uEPSC amplitudes induced by two-photon uncaging of MNI-glutamate onto a single spine head under the baseline condition (black) and after TCM application (gray). (D) Paired *t* test; 73/4; *T*_72_ = 4.98, ****P < 0.0001. Lines in (F) indicate the mixed Gaussian fit of the uEPSC amplitude distribution.

We used two-photon glutamate uncaging to activate AMPARs at single spines by setting the uncaging laser power according to the distance from the spine of interest to the surface of the slice (*59*). We filled L2/3 pyramidal neurons in P30 visual cortex slices with the fluorescent dye Alexa 594 and imaged them with two-photon laser scanning microscopy. Brief uncaging pulses directed next to dendritic spines located on secondary basal dendrites evoked inward currents measured at −70 mV (Fig. 10, A and B). Uncaging at different spots with a similar distance to the spine surface evoked uncaged EPSCs (uEPSCs) that varied in size dependent on the uncaging location with a maximum when the spot presumably faced the PSD (Fig. 10B). After a set of uncaging measurements (ranging from 16 to 30 spines) (Fig. 10C), mEPSCs were measured under the baseline condition. TCM was then added to the extracellular solution under the test condition and allowed to equilibrate for 5 min. Afterward, mEPSCs were recorded again, and the same set of dendritic spines was probed with uncaging pulses.

TCM application increased the frequency of mEPSCs (from 2.8 ± 0.2 Hz in the baseline measurement to 11.9 ± 2.7 Hz in TCM, *P* < 0.05, *n* = 4, paired *t* test), while in interleaved control experiments, there was no change in mEPSC frequency (from 3.3 ± 0.4 Hz in the baseline measurement to 2.9 ± 0.3 Hz afterward, *P* = 0.24, *n* = 4, paired *t* test). The average amplitude of the mEPSCs was not significantly changed under either TCM or control conditions [in TCM, baseline amplitudes were −9.6 ± 0.4 and −11.0 ± 1.0 pA after TCM addition (*P* = 0.24, *n* = 4, paired *t* test); under the control condition, baseline amplitudes were −9.9 ± 0.6 and −8.3 ± 0.8 pA afterward (*P* = 0.058, *n* = 4, paired *t* test)]. This internal control echoed our observation that TCM affected primarily the frequency and not the amplitude of miniature synaptic transmission (Fig. 3).

Sixty-six of 72 spines responded to the two-photon uncaged glutamate with a uEPSC >1.5 pA. The uncaging responses were enhanced in the presence of TCM. In the baseline period, the uEPSC increased by twofold after TCM application (Fig. 10D). This enhancement cannot be totally accounted for by the increase in mEPSC amplitude (Fig. 3D); thus, it was likely due to the recruitment of previously idled AMPARs by TCM at the idle-able transmission sites. In interleaved control experiments without TCM application, the uEPSC amplitudes decreased over time (fig. S10), further consistent with the TCM effect to recover previously idled transmission sites. The TCM-induced amplitude increase was independent of the initial uEPSC amplitude (Fig. 10, D and E), consistent with the mixed model of transmission sites. The baseline uEPSC amplitudes were best fitted by a multiple Gaussian distribution with at least three distinct peaks (Fig. 10F), suggesting that some spine heads are likely occupied by multiple effective transmission sites during the baseline (Fig. 9D). TCM shifted the distribution with a smaller proportion of the smaller amplitudes and extended the distribution with a higher proportion of bigger amplitudes (Fig. 10F), again consistent with the mixed model simulation (Fig. 9F). Thus, the two-photon uncaging results were supportive of the mixed model that distinct transmission sites, presumably idle-able and silenceable, coexist on single spines.

## DISCUSSION

We discovered two distinct transmission sites within the spines of L2/3 pyramidal neurons in the visual cortex. These sites had specific roles: One primarily expressed experience-dependent strengthening via silent synapse unsilencing, which we measured with evoked synaptic transmission (referred to as silenceable transmission sites), while the other one expressed synaptic transmission homeostasis measured through the constant frequency of miniature synaptic transmission (referred to as idle-able transmission sites). Although these two transmission sites are separate, they likely both contribute to evoked synaptic transmission and miniature synaptic transmission in varying degrees at different synapses in the brain (*3*, *38*, *60*).

In mature neurons, the idle-able transmission sites are predominantly in an idled state, which renders them nonresponsive to glutamate release. However, they became responsive when desensitization inhibitors like TCM or CTZ were applied. During development, the fraction of idled transmission sites increased. This process, referred to as synapse idling plasticity, maintained the frequency of miniature synaptic transmission constant. The constant properties of miniature synaptic transmission stood in contrast to the increased synaptic strength of evoked glutamatergic transmission during development. We propose that synapse idling plasticity responds to the maturation of silent synapses during developmental critical periods. This mechanism helps to maintain synaptic homeostasis while the cortical neural network undergoes experience-dependent strengthening.

### mEPSC frequency was governed both pre- and postsynaptically

The AMPAR desensitization inhibitors TCM and CTZ increased mEPSC frequency several-fold while having only a modest effect on quantal amplitude. Our results indicate that TCM primarily boosted frequency by releasing AMPARs from a nonresponsive state at specific transmission sites. We arrived at this conclusion on the basis of the following observations: (i) TCM affected AMPAR miniature EPSCs but not NMDAR ones. (ii) The increase in AMPAR responses caused by TCM of two-photon glutamate uncaging was of comparable size to the frequency increase. Notably, this procedure bypassed presynaptic glutamate release and indicated the effect size to be mediated postsynaptically. (iii) Different chemical classes of inhibitors of desensitization affected mEPSC amplitude and frequency differently, indicating a mechanism distinct from desensitization. (iv) TCM enhanced frequency more at P30 compared to P16, with a similar increase in amplitude, indicating plasticity in the amount of idling. (v) The amplitude distribution of mEPSCs contained two distinct populations that differed in relative size between P16 and P30, and only one was prone to idling. We excluded the notion that the frequency increase by TCM was caused by the concomitant amplitude increase. Thus, while a fraction of mEPSC may remain undetected because of recording noise, more than two-thirds of spontaneous vesicle fusions were undetected because AMPARs were unresponsive at idled transmission sites under basal conditions. The fraction of idled transmission sites differed at the two developmental time points, indicating a developmental regulation, referred to as synapse idling plasticity that was regulating the frequency of mEPSCs and contributing to the constant frequency of mEPSCs during development.

The effect of CTZ to increase mEPSC frequency has been previously attributed to a presynaptic mechanism, enhancing release probability in specific synaptic contexts such as autapses of hippocampal cultures or Calyx of Held synapses (*3*, *37*). However, we found no evidence that the related desensitization blocker TCM increased release probability or vesicle fusogenicity in L2/3 pyramidal neurons. In addition, CTZ was reported to not affect release probability at climbing fiber synapses or synapses of CA1 pyramidal neurons (*61*, *62*). Thus, CTZ’s presynaptic effect may vary among synapses, while TCM appears not to have such a presynaptic effect. No mechanism has been described so far that can explain such a large increase in vesicle fusogenicity or release probability induced by CTZ. Given the similar effect size of CTZ and TCM, we suggest that both drugs primarily enhance mEPSC frequency by making previously unresponsive AMPARs at idled transmission sites responsive.

TCM and CTZ increase the median effective concentration of AMPARs for glutamate by affecting primarily the *k*_off_ (*63*). This effect is consistent with the similar amplitude increase of TCM for both evoked and miniature EPSCs. However, it is inconsistent with the preferential frequency increase as the increase in quantal content was bigger than that in quantal size. If TCM would have caused the notion that glutamate of a single vesicle activated more AMPARs, the amplitude increase would have dominated. Furthermore, the more punctual release of glutamate from a single vesicle compared to two-photon glutamate uncaging would have caused a bigger amplitude increase because the receptor density is higher at the synapse than it is extrasynaptically (*24*, *64*). Given that the amplitude increase had a relatively small effect on frequency increase by lifting events above the detection threshold, the dominating effect that we observed was the unidling of previously not responding transmission sites.

Therefore, besides mEPSC frequency being influenced by factors such as synapse number (*15*), presynaptic mechanisms that enhance vesicle fusion (*65*, *66*), and postsynaptic mechanism in silent synapses (*11*, *35*), we discovered a potent regulation at the postsynapse, the idling of transmission sites that matches the potency of previously identified presynaptic mechanisms (Fig. 3). Synapse idling challenges the common assumption that changes in mEPSC frequency likely refer to presynaptic mechanisms but provides a potential explanation for previously unresolved observations in which changes in release probability contradicted changes (or thereof) in mEPSC frequency (*67*, *68*).

### Idle-able and silenceable transmission sites operated in parallel on single spines

Our results indicate that idle-able and silenceable transmission sites are distinct. This conclusion is supported by the following observations. The strength of evoked AMPAR-mediated transmission increased during development, while the amplitude and frequency of miniature synaptic transmission remained constant. This difference was attributed to idling synapse plasticity, which increased the fraction of idled transmission sites for miniature synaptic transmission during development. Two distinct populations of mEPSCs were identified by the analysis of the mEPSC amplitude distribution, and the fraction of one increased after TCM application, which we interpret as idled transmission sites that were unidled by TCM. In contrast, TCM had comparatively little effect on the strength of evoked AMPAR-mediated synaptic transmission in visual cortex L2/3 pyramidal neurons, indicating separable pools of AMPARs for the two modes of transmission. TCM increased the amplitude of evoked AMPAR-mediated synaptic transmission by ~40% by blocking acute desensitization and broadening the EPSC. A similar increase was observed for mEPSC amplitudes. In contrast, TCM increased the frequency of mEPSCs several-fold, revealing the high fraction of idled miniature synaptic transmission sites. These results suggest that not all miniature transmission and evoked transmission occur through a single mode using the same presynaptic release sites and postsynaptic AMPARs. It is plausible that miniature transmission sites are shared by evoked transmission. Whether the TCM-resistant population in the mEPSC amplitude histogram corresponds to the transmission sites that we refer to as silenceable needs further investigation. Notably, in PSD-95–deficient neurons, not only silent synapse maturation is impaired, which causes the persistence of silent synapses during development, but also mEPSC frequency is reduced, indicating that PSD-95 regulates both transmission sites or that the frequency homeostasis is impaired without PSD-95 and that the reduced mEPSC frequency is caused by impaired silent synapse unsilencing (*11*, *34*).

We simulated the effects of TCM on AMPAR unidling when nearly all AMPARs within a spine tip are exposed to glutamate, as in two-photon glutamate uncaging experiments. We considered two models: a uniform model, where all AMPARs within a given spine were in the same state, while silenceable and idle-able transmission sites were on different spines. This assumption was necessary because, in the uniform model, glutamate release from synaptic vesicles can reach any AMPAR in the spine. Given our observation of an increase in frequency with TCM, mixing AMPAR states within a spine would have resulted in a preference of amplitude increases. In contrast, the mixed model assumed that silenceable and idle-able transmission sites were on the same spine, but a synaptic vesicle fusion could only activate one cluster of AMPARs with a shared functional state. Experimental results from two-photon glutamate uncaging supported the mixed model. Specifically, in the uniform model, we would have expected a high fraction of unresponsive spines to account for the increase in mEPSC frequency, which was not observed. In addition, we primarily observed increases in previously responsive spines, consistent with the mixed model rather than the uniform one. Last, the amplitude distribution shifted to larger amplitudes, as predicted by the mixed model but not the uniform one. These results suggest the existence of distinct transmission sites within single spines.

Our results extend the interpretation of previously described confined transmission sites in which release sites are aligned with postsynaptic AMPAR clusters to increase their proximity and estimation that the activation of AMPARs is spatially constrained to a 125-nm radius from the center of glutamate release (*25*, *69*). Thus, the identified AMPAR clusters in the range of 70 nm, which might be positioned opposite of receptor clusters to ensure that their activation might represent individual transmission sites (*70*). Whether silenceable and idle-able transmission sites operate in such a configuration will need further research.

Our results also align with previous reports indicating heterogeneity in presynaptic release sites and postsynaptic receptors that are differentially involved in miniature synaptic transmission and evoked synaptic transmission (*27*, *71*). While miniature release and evoked release have been reported at the same hippocampal synapse (*72*), our findings suggest that entire transmission sites at single synapses are distinct with idle-able and silenceable transmission sites operating in parallel within a single synapse, as outlined in the mixed model. However, this implies that previous observations of postsynaptic receptor involvement need to be reconsidered with distinct transmission sites. For example, it was reported that a single vesicle does not saturate postsynaptic receptors in a synapse (*26*). In the mixed model, one synaptic vesicle only reaches a single receptor cluster in a single transmission site. As such, multiquantal release might engage multiple transmission sites within a single spine, potentially also involving idle-able transmission sites. However, we did not observe this because our conditions of evoked synaptic transmission favored uniquantal release and thus only triggered release at a single transmission site (*73*).

### Idling of AMPARs was distinct from their desensitization

The shared function of CTZ and TCM in inhibiting AMPAR desensitization suggests that the idle state of the transmission sites may result from AMPARs persistently locked in a desensitized state. Low glutamate and the binding of a single glutamate favor the transition into a desensitized state (*41*). This would imply that glutamate is kept low at idle-able transmission sites but not at silenceable ones. However, desensitized AMPARs are replaced quickly from synapses, rendering it unlikely that they reside in a chronically desensitized state (*70*, *74*). Our finding that different classes of desensitization inhibitors differentially restored the unresponsive transmission sites also argues for a distinct mechanism. While the different classes of inhibitors share the function to inhibit the transition of AMPARs into the desensitized state by stabilizing the ligand binding domain interface, they have different contact sites and CTZ and TCM might act in addition through a separate site compared to inhibitors not able to unidle AMPARs (*43*, *75*). In support for two separate actions of CTZ, CTZ inhibition of desensitization is almost instantaneous, while its effect on enhancing AMPAR currents develops slowly within 10th of seconds (*76*). Furthermore, while CTZ enhanced AMPAR currents evoked by kainate, a nondesensitizing partial agonist, aniracetam does not, indicating differential actions of the two drugs (*77*). Whether the different contact sites or a distinct binding site for CTZ and TCM is responsible for the unidling will need further investigation. Notably, aniracetam increased frequency at P16 but not at P30. This frequency increase was lower than that of TCM and CTZ, which indicated a lower potency of aniracetam. However, this result also suggested that the susceptibility to be released from the idled state differed at P16 and P30. A possible explanation would be that posttranslational modifications of AMPARs or different association with auxiliary subunits at P16 versus P30 regulates this susceptibility. These features suggest that the unresponsive state in idled transmission sites might not belong to the gating cycle of AMPA receptors but rather constitutes a novel undefined state that keeps them idle.

### Synapse idling plasticity contributed to the homeostasis of miniature synaptic transmission

Contrary to current synaptic concepts, we found that two types of synaptic plasticity are expressed at different transmission sites. Specifically, the homeostasis of miniature synaptic transmission occurs over the critical period of visual cortex development at idle-able transmission sites, while synaptic strengthening via experience-dependent plasticity occurs at silenceable transmission sites within the same synapse. Furthermore, we found that the idling plasticity responsible for maintaining mEPSC homeostasis was linked to the maturation of silent synapses despite the two modes of transmission occurring at different transmission sites. The following observations supported our conclusions. The mEPSC frequency stabilized after P10, as did mEPSC amplitude in visual cortex L2/3 neurons (Fig. 2). This phenomenon is also observed in pyramidal neurons across other layers in the visual cortex and in hippocampal cultures (*15*, *19*, *78*). This frequency homeostasis results from a combination of activity-dependent pruning during a critical period and an increase in the idling of transmission sites, namely the idle-able transmission sites (Fig. 3) (*15*). Essentially, during this developmental phase, silent synapses mature, leading to an increase in synaptic strength of evoked transmission. Concurrently, the fraction of idled transmission sites also increased, causing the homeostasis of mEPSC frequency.

In summary, we propose that synapse idling plasticity and silent synapse maturation are two forms of plasticity that operate at distinct parallel transmissions sites. They may collaborate to establish cortical neural networks that can undergo experience-dependent synapse refinement to eventually reach a stable state after synapse idling.

## MATERIALS AND METHODS

### Chemicals

Chemicals were purchased from Sigma-Aldrich, if not otherwise specified. CTZ, aniracetam, and Cx614 were purchased from MedChemExpress. Tetrodotoxin (TTX), NBQX, APV, and picrotoxin were purchased from HelloBio.

### Mice

C57Bl6 mice were group housed, two to five per standard cage (33 by 17 cm), under a 12-hour light/dark cycle and with food and water ad libitum. All procedures were performed during the light cycle by strictly following the procedures approved by the animal care and use committees and governmental agencies of the listed institutions (Institutional Animal Care and Use Committee no. 24055113, T23-34).

### Visual cortex slice preparation

Coronal visual cortical slices (300 μm) from P10, P14, P16 ± 1 day, P20, or P30 ± 2 days mice were sliced with a vibratome in ice-cold sucrose (168 mM sucrose, 25 mM NaCl, 1.9 mM KCl, 10 mM MgSO_4_, 26 mM NaHCO_3_, 1.2 mM NaH_2_PO_4_, and 25 mM d-glucose) or *N*-methyl-d-glucamine (NMDG) cutting buffer (135 mM NMDG/HCl, 1 mM KCl, 1.5 mM MgCl_2_, 20 mM choline/HCO_3_, 1.2 mM KH_2_PO_4_, 10 mM d-glucose, and 0.5 mM CaCl_2_). Slices were recovered at 35°C for 20 min in standard ACSF [119 mM NaCl, 26 mM NaHCO_3_, 20 mM d-glucose, 2.5 mM KCl, 1 mM NaH_2_PO_4_, 1.3 mM MgSO_4_, and 2.5 mM CaCl_2_, saturated with carbogen, 95% (v/v) O_2_, and 5% (v/v) CO_2_] and then stored in carbogenated ACSF at room temperature until further use. For two-photon glutamate uncaging, the brain was rapidly removed into ice-cold ACSF consisting of 119 mM NaCl, 4.2 mM KCl, 26 mM NaHCO_3_, 1 mM NaH_2_PO_4_, 1.2 mM CaCl_2_, 0.7 mM MgCl_2_, 1.3 mM Na-ascorbate, and 3 mM Na-pyruvate. The brain was fixed to a slicing platform, and 270-μm-thick coronal slices were made using a VT-1200S vibratome (Leica). Slices were collected and transferred to a warm (34°C) ACSF solution until use.

### Slice electrophysiology

Standard whole-cell voltage-clamp recordings were carried out at 31° to 32°C in a recording chamber, which was perfused (2 ml/min) with ACSF. L2/3 pyramidal neurons were visually identified with infrared-differential interference contrast microscopy. Glass pipettes (3 to 5 megohms) were filled with a Cs-based internal solution (117.5 mM CsMeSO_3_, 10 mM Hepes, 17.8 mM CsCl, 10 mM TEA-Cl, 0.25 mM EGTA, 10 mM d-glucose, 2 mM MgCl_2_, 4 mM Na-ATP, 0.3 mM Na-GTP, pH 7) to record A/N ratios and for the failure analysis and a low-Cl^−^ internal solution (133 mM CsMeSO_3_, 10 mM Hepes, 10 mM TEA-OH, 0.25 mM EGTA, 10 mM d-glucose, 2 mM MgCl_2_, 5 mM QX314-Cl, 4 mM Na-ATP, and 0.3 mM Na-GTP, pH 7) to record AMPA and NMDA receptor synaptic currents, evoked with theta-glass bipolar electrodes filled with ACSF and placed in L4. Picrotoxin (50 μM) was added to ACSF to block γ-aminobutyric acid (GABA)–mediated synaptic transmission, unless otherwise stated. The input and series resistance were monitored throughout the recording by applying a short hyperpolarizing voltage step before synaptic stimulation. Only cells with a series resistance smaller than 30 megohms and changes of series and input resistance of less than 20% were used for analysis. Data were filtered at 3 kHz and collected with custom routines in Igor (Wavemetrics) using an ELC-03XS amplifier (NPI) and digitized at 10 kHz with an ITC-18 (HEKA).

### Recording and two-photon glutamate uncaging

Voltage-clamp recordings were made using pipettes (PG52151-4; World Precision Instruments) pulled (P-97; Sutter) to have an open-tip resistance ranging from 3 to 5 megohms when filled with an internal solution consisting of 130 mM Cs-methanesulfonate, 5 mM NaCl, 10 mM Hepes, 5 mM TEA-Cl, 4 mM MgCl_2_, 4 mM Na-ATP, 0.4 mM Na-GTP, 10 mM Na-phosphocreatine, 0.1 mM EGTA, 0.03 mM Alexa 594 (pH 7.35) with CsOH at a measured osmolality of 300 mosmol and a measured junction potential of −8 mV relative to ACSF. Cells were held at −60 mV, and currents were recorded with a Multiclamp 700B amplifier (Molecular Devices) controlled by Prairieview 5.4 software (Bruker). Current measurements were sampled at 10 kHz and filtered at 3 kHz. Recording ACSF consisted of 119 mM NaCl, 2.5 mM KCl, 26 mM NaHCO_3_, 1 mM NaH_2_PO_4_, 2.5 mM CaCl_2_, 1.3 mM MgSO_4_, 1.3 mM Na-ascorbate, and 3 mM Na-pyruvate, supplemented with 0.5 μM TTX (Alamone Labs) to block sodium channels, 50 μM picrotoxin (Abcam) to block GABA_A_ receptors, and 2.5 mM MNI-glutamate (Tocris). The extracellular solution was continuously perfused at a rate of 2 ml/min controlled with a PPS2 peristaltic pump (Multichannel Systems), and the temperature was maintained at 30° to 31°C with an inline heater (ALA Scientific) controlled with a TC-20 feedback controller (NPI).

Neurons were visualized with a two-photon imaging system (Bruker) using an excitation wavelength of 840 nm (Coherent Ultra II). MNI-glutamate uncaging was performed by directing a second laser (Coherent Ultra II) tuned to 720 nm next to a dendritic spine. Uncaging laser duration was set to 0.5 ms, and power was set on the basis of the depth in the slice to a level that would photobleach the Alexa 594 dye in the dendritic spine by ~40% (*59*). The excitation laser filled the back aperture of the Olympus 60× 1.0–numerical aperture objective. The point spread function (full-width at half-maximal intensity) was measured to be 0.52 μm in the *x*-*y* plane and 2.23 μm in the *z* axis using 0.085-μm-diameter fluorescent beads (Polysciences). Figure 10 (A and B) shows an example uncaging experiment where the uncaging spot targeted to different points around a spine led to different uEPSC response sizes.

After establishing a whole-cell recording, dye was allowed to fill the cell for at least 15 min before imaging. Before uncaging, we recorded for 200 s (20 sweeps of 10 s) to measure mEPSCs. Dendritic spines on basal dendrites were targeted for glutamate uncaging experiments (mean distance from the soma, 57 ± 2 μm; *n* = 80 spines). Four uncaging repetitions were performed at 6-s intervals on each spine in the set. Following baseline uncaging, the extracellular solution was supplemented with 500 μM TCM (test condition) or not (control condition) and allowed to recirculate for 5 min before further measurements. After solution exchange, the same set of dendritic spines was tested with uncaging, and spontaneous EPSCs were measured as before. Responses to MNI-glutamate uncaging were measured as the peak of the response after averaging the four repetitions for each spine.

### Miniature EPSC recording

mEPSCs were recorded in the presence of 0.5 μM TTX and picrotoxin (50 μM) to block voltage-gated Na^+^ channels and GABA_A_ receptors, respectively. Events were analyzed with Minianalysis (Synaptosoft). mEPSCs were automatically detected using the program with an 8-pA amplitude threshold or 5 pA when indicated, and all events were verified visually. For mEPSCs, more than 400 events of each cell were recorded, sorted by mEPSC amplitude size, and binned in 20 bins. Averages of bin values from different cells were plotted as cumulative probability plots. NMDAR-mediated mEPSCs were recorded at −40 mV in ACSF containing 50 μM picrotoxin, 0.5 μM TTX, 10 μM NBQX, and 0.2 mM MgCl_2_. APV (50 μM) was added at the end of the experiment. Five trials of 10-s recording were used for analysis for each condition. The baseline was subtracted using the Baseline Fitting package in Igor, and the area under the curve was calculated as the measurement of total charge. mEPSCs during two-photon glutamate uncaging were detected with a template-matching algorithm (*79*).

### Current intensity recording

For examining the relationship between EPSC amplitude and stimulus intensity, picrotoxin was omitted to prevent epileptiform responses in the slices when a high level of stimulation was used. Stimulation was adjusted to trigger an EPSC of ~50 pA. Then, 2×, 3×, 4×, and 5× of the original stimulation strength were used to record the relationship between stimulation strength and EPSC amplitude.

### AMPA/NMDA receptor ratio

The AMPAR component was measured as a peak value at *V*_*h*_ = −60 mV, and the NMDA receptor component was measured at *V*_*h*_ = +40 mV at 60 ms after the stimulation. 2-Chloroadenosine (1 μM) was used to prevent polysynaptic activity (*12*).

### Paired-pulse ratio

Cells were held at −60 mV for the recording of paired pulses of AMPAR-EPSCs or −40 mV for the recording of paired pulses of NMDAR-EPSCs (with 10 μM NBQX). Paired stimuli with interstimulus intervals of 50 and 100 ms were delivered every 5 s. PPR was measured as the ratio of the second EPSC amplitude to the first one.

### Synaptic failure analysis

The stimulation strength was adjusted such that ∼50% of the trials elicited AMPAR responses at a *V*_*h*_ = −60 mV. At a *V*_*h*_ = +40 mV, we then measured a composite response mediated by AMPARs and NMDARs. For each trial, 30 to 50 sweeps at each holding potential were recorded. The % of silent synapses was calculated by using the equation 1 − ln(*F*_−60_)/ln(*F*_+40_), in which *F*_−60_ is the failure rate at −60 mV, and *F*_+40_ is the failure rate at +40 mV (*11*, *12*, *15*).

### Simulation of failure analysis

We simulated synaptic events for a unitary connection between L4-to-L2/3 pyramidal neurons with the binomial model

P(k;n;p)=nkpk(1−p)(n−k)

*P* is the probability of *k* synaptic vesicle fusions at *n* synapses with the synaptic release probability *p* for a single action potential. We varied *k* for the unitary synaptic connections between 3 and 5 based on a previous report (*17*). For synaptic transmission, at *V*_*h*_ = +40 mV, the synaptic release probability was varied between 0.3 and 0.5 with the same value for the *n* synapses of one cell (*28*). To account for the % of silent synapses, at *V*_*h*_ = −60 mV, the synaptic release probability was multiplied by 1 minus the % of silent synapses. For P16, the % of silent synapses was varied per L2/3 pyramidal neuron with a normal distribution around the mean of 0.5 ± 0.15, whereas for P30, it was around the mean of 0.20 ± 0.15 (*11*).

### Calculating the possible contribution of mEPSC amplitude increase on frequency increase

Minirecordings with low baseline noise that allow identification of miniature events starting from 5 pA were used for the analysis. The frequency of the events with a 5-pA detection threshold was determined as described above. For events in TCM, the amplitude was multiplied by the scalar of 0.7 to equalize the TCM-mediated upscaling. The number of events that fall below the 5-pA detection threshold was determined, and their contribution to the frequency in TCM was calculated by dividing by the total number of events in TCM times the frequency. This value represented the maximal possible frequency contribution of amplitude scaling by TCM to its frequency increase. In analogy, the remaining events for the 8- or 10-pA threshold were determined for ACSF and TCM conditions to calculate the corresponding frequency of events. The corresponding maximal possible frequency contribution of amplitude scaling by TCM for the 8- or 10-pA detection threshold was then calculated as described for the 5-pA threshold.

### Fitting of mEPSC amplitudes

Minirecordings with low baseline noise that allow identification of miniature events starting from 5 pA were used for fitting. The recordings from a cell in ACSF were first fitted with a single gamma distribution

χ∼Γ(α,β)

where α is the shape factor, and β is the scale factor. The upper bound of α was set at 40. The fitted α and β were then used as the initial guesses for a mixed gamma distribution fit with two components, with the initial guess of the weight (κ) at 0.5

$$\chi ∼\kappa *\Gamma_{1}\left(\alpha_{1},\beta_{1}\right)+\left(1-\kappa \right)*\Gamma_{2}\left(\alpha_{2},\beta_{2}\right)$$

The higher value of the shape factor is assigned as α_1_, and the weight is assigned accordingly.

The recordings from the same cell in TCM were fitted with a single gamma distribution

$$\chi_{T}∼\Gamma_{T}\left(\alpha_{T},\beta_{T}\right)$$

For the mixed gamma distribution fit, α_1_ and α_2_ were taken from the fitted parameters of ACSF recordings, leaving the weight and the shape factors free for fitting

$$\chi_{T}∼\kappa_{T}*\Gamma_{1T}\left(\alpha_{1},\beta_{1T}\right)+\left(1-\kappa_{T}\right)*\Gamma_{2T}\left(\alpha_{2T},\beta_{2T}\right)$$

The goodness of fit of the single gamma fit and mixed gamma fit was assessed using the likelihood ratio test, where the null hypothesis was that the single gamma fit was a better fit for the data. The *P* value approaching 0 indicated that there was strong evidence suggesting that the mixed gamma model fitted the data better than the single gamma model.

### Use-dependent block of NMDARs by MK-801

Neurons were clamped at +40 mV. Evoked NMDAR-EPSCs were recorded in the presence of 5 μM NBQX (AMPAR antagonist) and 5 μM MK-801 (use-dependent NMDAR blocker). Fifty sweeps were recorded, and the intertrial interval was 10 s. The EPSC amplitude was normalized for each cell to the first EPSC in MK-801. Progressive blockade of EPSCs was fitted to a double-exponential function. TCM (500 μM) was included to test the potential presynaptic effect of the drug (*49*, *68*).

### Viral vectors

An AAV with an shRNA against PSD-95 was used to silence PSD-95 expression. An AAV with an shRNA against luciferase or with an expression cassette for enhanced green fluorescent protein only was used as a control for shRNA expression or AAV transduction, respectively (*11*, *12*, *15*). AAVs were produced according to described procedures, pseudotyped with the capsid for AAV8, and purified by iodixanol gradient centrifugation (*80*).

### In vivo AAV injection

For P0 mice AAV delivery, P0-P1 mouse pups were anesthetized on ice for 5 min and immobilized with a holder on the injection table, where the surface temperature was kept at 4°C. The intersection of the sagittal suture and lambdoid suture (lambda) was visually identified through the skin. Injections of AAV (13.4 to 67 nl each, 23 nl/s) were delivered at two positions bilaterally into V1 [from lambda: AP (anterior/posterior) +0.1 mm, ML (medial/lateral) ±1.25 mm, and DV (dorsal/ventral) −0.8 mm and AP +0.3 mm, ML ±1.8 mm, and DV −0.8 mm] using a glass capillary with a Nanoject II microinjector (Drummond) (*11*, *12*).

### Fluorescent dye microinjection

Mice were transcardially perfused with ice-cold 1% paraformaldehyde (PFA) in 0.1 M phosphate buffer, followed by 4% PFA and 0.125% glutaraldehyde in 0.1 M phosphate buffer. Brains were removed and postfixed in 4% PFA and 0.125% glutaraldehyde in 0.1 M phosphate buffer for 12 to 14 hours at 4°C. Following postfix, the brains were transferred into 0.1 M phosphate-buffered saline and sectioned into 250-μm-thick slices using a VT1200S vibratome (Leica). Cells were filled shortly after slicing (within 4 hours). Cells within the visual cortex L2/3 were impaled with a fine micropipette containing 5 mM Alexa 594 hydrazide (Invitrogen) and injected with a negative current of 1 to 10 nA until dendrites and spines were filled.

### Confocal imaging

Images were captured with a Leica TCS SP5 confocal microscope equipped with Leica Application Suite software (Leica). Individually filled neurons were visualized with a ×63 oil immersion objective for final verification. Individual dendritic segments of secondary basal dendrites were focused on and scanned at 0.69-μm intervals along the *z* axis to obtain a *z*-stack. Analyses were performed on two-dimensional projection images using ImageJ (National Institutes of Health). For each neuron, one to four (2.1 average) dendritic segments of ~20 μm in length were analyzed. For each group, four to eight cells per mouse were analyzed. *N* is the number of cells with the dendritic segments averaged for one data point.

### BS^3^ cross-linking and immunoblot analysis

Acute brain slices were prepared in NMDG cutting solution. Slices were incubated in ACSF containing 0.5 μM TTX and 50 μM picrotoxin for 5 min. Then, the solutions were replaced with ASCF (with TTX and picrotoxin) containing the specific drugs and BS^3^ (1 mg/ml, Thermo Fisher Scientific). After 30 min, the slices were washed twice with chilled ACSF and incubated on ice for 30 min. Tris (100 mM) was used to quench the BS^3^. Then, slices were sonicated in ACSF containing 1% SDS. Protein concentration was determined by the BCA kit (Pierce). Twelve micrograms of total protein was loaded into each sample for SDS–polyacrylamide gel electrophoresis with immunoblot analysis. Bands were decorated by vGluT1 (N28/9, NeuroMab), GluA2 (L21/32, NeuroMab), and actin (AC-40, Thermo Fisher Scientific) antibodies, visualized with the secondary antibody goat anti-mouse IR800 (Li-COR Biosciences), and quantified with an infrared fluorescence scanner (*12*, *53*).

### Simulation

The simulation framework was designed to simulate a population of spine heads, each with a random number of transmission sites within a range and each site having a random identity among two transmission types (silenceable and idle-able). Each type had two transmission modes (silent, unsilenced or idled, unidled) based on a given probabilistic weight, with each unitary response size chosen randomly from an array of 50 by 50 generated from a normal distribution with the mean of −4 pA and variance of 1. In the uniform model (Fig. 9A), all transmission sites on a single spine head were of the same type. The probability of the transmission type and mode was assigned according to the predictions from the electrophysiology results. In the mixed model (Fig. 9B), the number of silenceable transmission sites per spine head was randomly determined within a specified range, and their modes were assigned on the basis of probabilities predicted from the electrophysiology results. This further determined the number of idle-able transmission sites per spine head and the probabilities of either mode. The TCM treatment switched the idled idle-able transmission site from a noneffective state to an effective state of transmission and did not influence the state of transmission at other transmission sites.

### Data analysis

Statistical tests were performed using GraphPad Prism 7 (GraphPad Software, La Jolla, CA). Biological replicates were used with *n*, the number of neurons, and *m*, the number of animals, as described in figure legends (*n*/*m*). Unless otherwise stated, Student’s *t* test or one-way analysis of variance (ANOVA; for multiple groups, including repeat measures where applicable) followed by Bonferroni’s multiple comparison test was used. Two-way ANOVA followed by Bonferroni’s multiple comparison test was used for experiments containing two independent variables. For Western blot data on different gels, one-way ANOVA with matched data and Geisser-Greenhouse correction, followed by Dunnett’s multiple comparison test, was used. All the data are represented as the means ± SEM. All conditions statistically different from the control are indicated: *P < 0.05; **P < 0.01; ***P < 0.001.
