# Supplementary material for: Distinct transmission sites within a synapse for strengthening and homeostasis
Source: Sci Adv. 2025 Apr 11;11(15):eads5750. doi: 10.1126/sciadv.ads5750 (PMC11988405; doi:10.1126/sciadv.ads5750)
Supplement: Supplementary file 1 — Figs. S1 to S10 Tables S1 and S2 [file sciadv.ads5750_sm.pdf]

Science Advances
AAAS

Supplementary Materials for

Distinct transmission sites within a synapse for strengthening and homeostasis

Yue Yang *et al.*

Corresponding author: Oliver M. Schlüter, schluter@pitt.edu

Sci. Adv. **11**, eads5750 (2025)
DOI: 10.1126/sciadv.ads5750

This PDF file includes:

Figs. S1 to S10
Tables S1 and S2

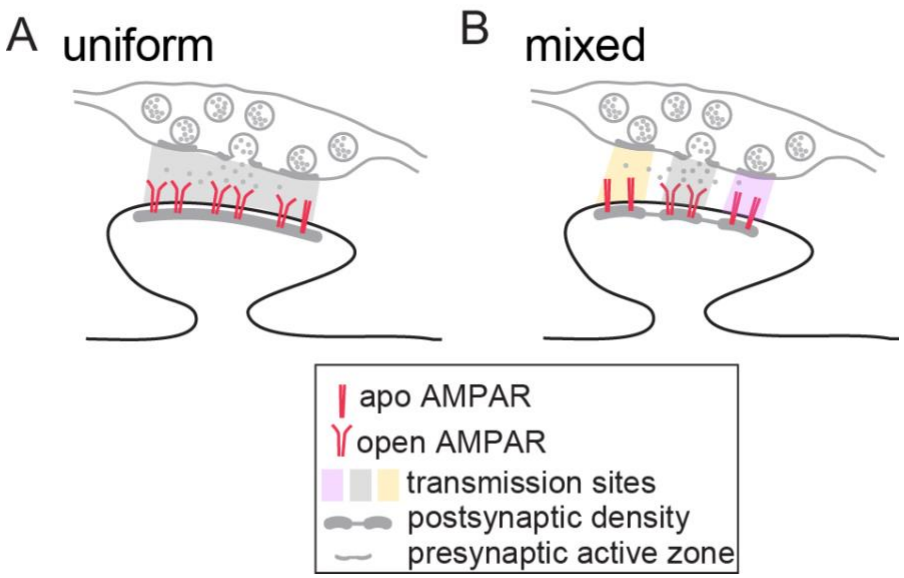

Fig. S1: Comparison of different transmission models at a spinous synapse.
(A) In the uniform model, synaptic vesicles fuse at different locations of the active zone and the released glutamate spreads over the entire synapse as it constitutes one transmission site (gray shade). Fusion at different locations will lead to glutamate concentration gradients that activates different but overlapping AMPARs. The state of the AMPARs at rest is uniform. (B) In the mixed model, different states of AMPARs exist at rest. Synaptic vesicle fusion only activates the AMPAR cluster directly opposing it. Thus, the vesicle location and postsynaptic AMPAR cluster are aligned, constituting different transmission sites (shaded colors).

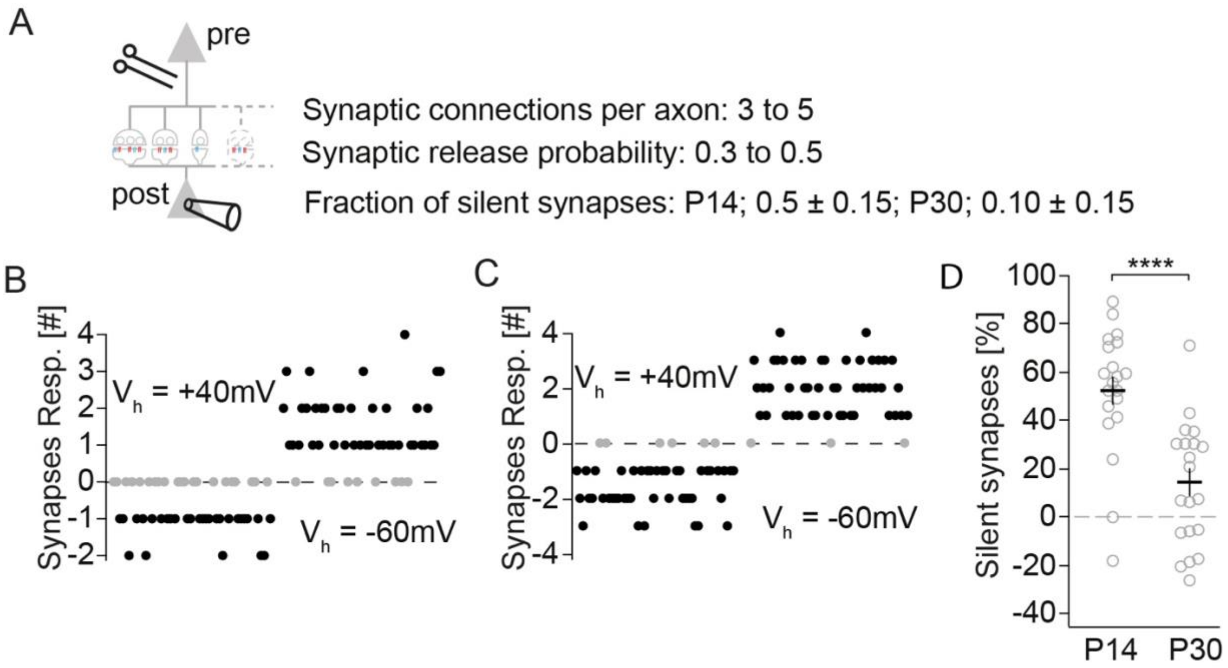

Fig. S2: Simulation of failure rate analysis for L4-to-L2/3 pyramidal neurons

(A) Scheme to illustrate a unitary connection between a L4 star pyramid and a L2/3 pyramidal neuron with 3 to 5 synaptic connections. L4 axon stimulation and L2/3 pyramidal neuron recording is depicted. Parameters to simulate synaptic transmission with a binominal model of synaptic vesicle fusion at a holding potential -60 mV with failures due to silent synapses and +40 mV without failures due to silent synapses. (B-C) Example cells at P14 (B) and P30 (C) with number (#) of synapses responding for each of 50 trials at indicated holding potentials (V_h) with successes (black) and failures (gray). (D) Summary graph of % silent synapses of 20 simulated neurons (open circles) at the indicated developmental time points. $T_{(38)} = 4.716$, ****$p < 0.0001$.

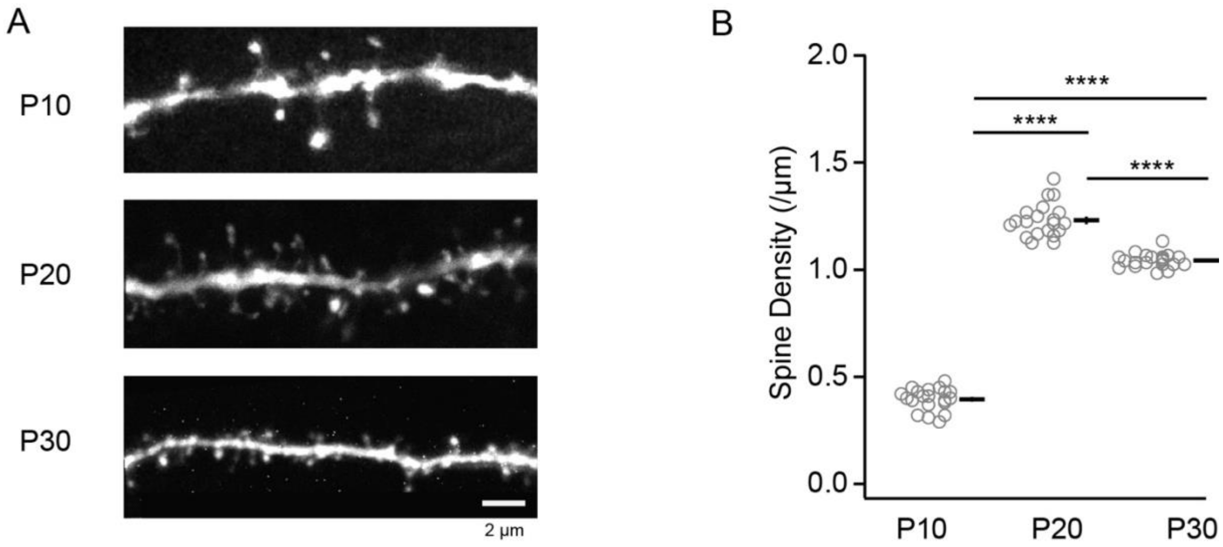

Fig. S3: Partial equilibration of increase in matured AMPAR-responsive synapses by synapse pruning.
(A) Sample images and (B) summary graph of secondary basal dendrites from P10 to P30 of visual cortex L2/3 pyramidal neurons filled with Alexa 594 dye in WT mice. Scale bar: 2 µm. P10 (20/3), P20 (20/5), P30 (20/3). 1-way ANOVA: $F_{(2,57)} = 1167$, $p < 0.0001$; P10 vs. P20 or P30: ****$p_{adj} < 0.0001$, P20 vs. P30: ****$p_{adj} < 0.0001$.

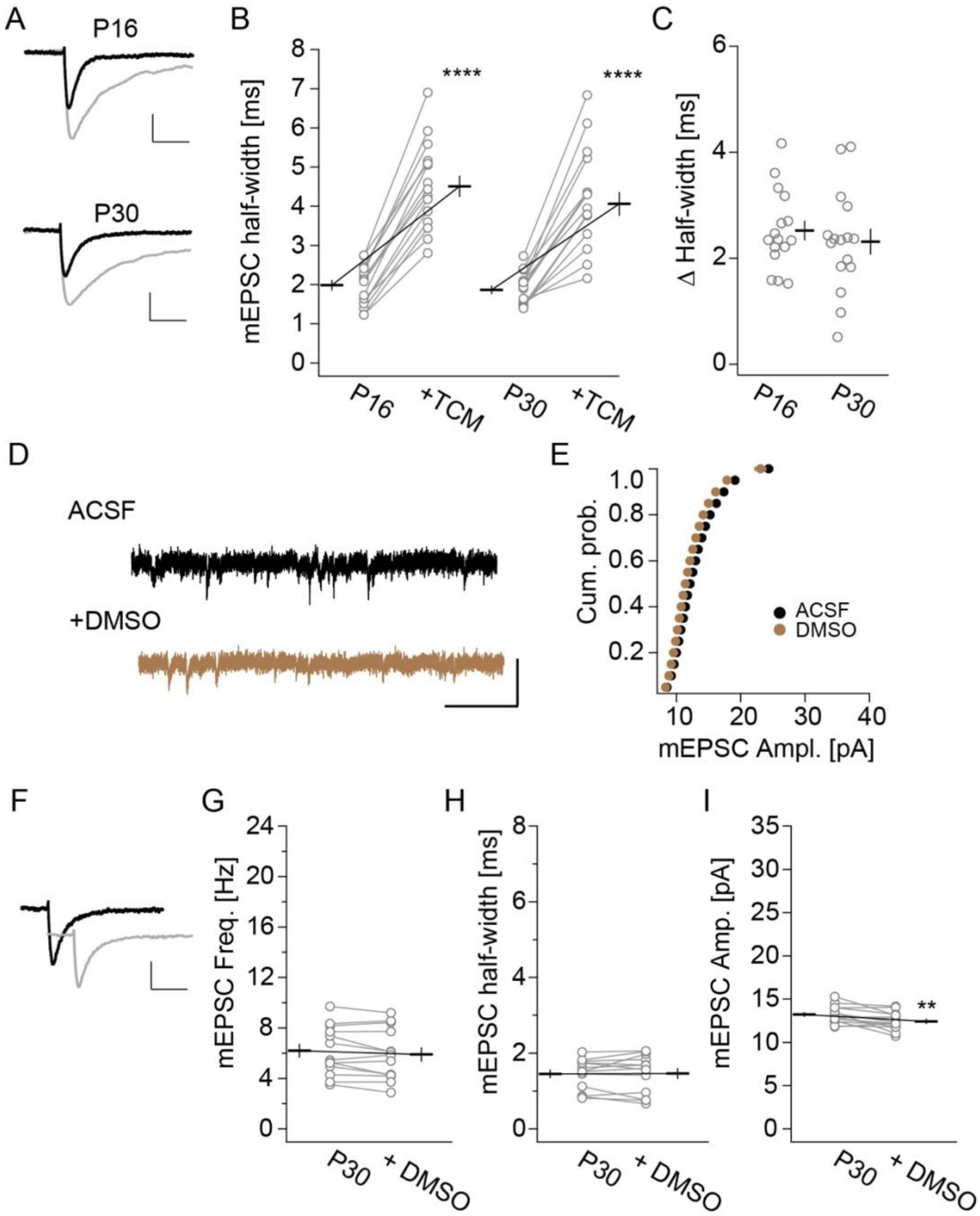

Fig. S4: Extended data for Figure 3.

(A) Sample traces of averaged mEPSCs at P16 and P30, in ACSF (black) and after TCM (gray). Scale bar: 5 pA, 10 ms. Summary graphs of mEPSC half-width before and after TCM at P16 or P30 (B) and half-width increase (ΔHalf-width; C). (B) 2-way ANOVA, $F_{(30, 30)} = 3.246$, $p < 0.001$; ACSF vs. TCM: $F_{(1,30)} = 252.3$, ****$p < 0.0001$; P16 vs. P30: $F_{(1,30)} = 0.61$, $p = 0.44$; interaction: $F_{(1,30)} = 0.4687$, $p = 0.50$. (C) $T_{(30)} = 0.685$, $p = 0.50$. (D) Example traces of AMPAR-mediated mEPSCs recorded in V1 L2/3 neurons from P30 mice before and after DMSO application. Scale bar: 200 ms and 20 pA. (E) Cumulative probability graph of AMPAR mEPSC amplitude. (F) Sample traces of averaged mEPSCs, in ACSF (black) and after DMSO (gray). Summary graphs of mEPSC frequency (G), half-width (H), and amplitude (I) before and after DMSO at P30. DMSO (13/4). (G) $T_{(12)} = 1.963$, $p = 0.073$. (H) $T_{(12)} = 0.1696$, $p = 0.87$. (I) $T_{(12)} = 3.194$, $p < 0.01$.

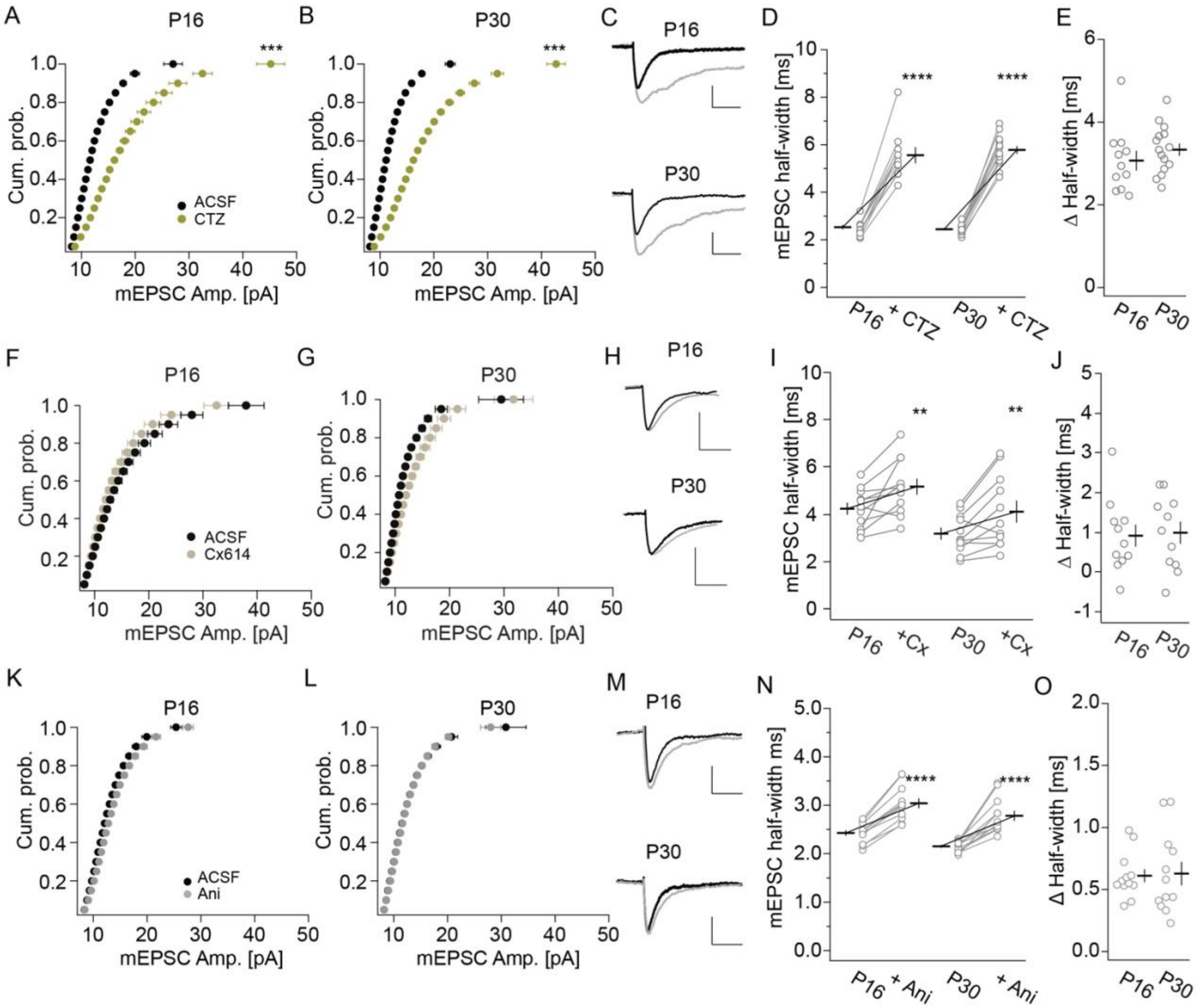

Fig. S5: Extended data for Figure 4.

(A, B) Cumulative probability plots for AMPAR mEPSC amplitude before and after CTZ at P16 (A) and P30 (B). KS-Test, $p < 0.001$. (C) Sample traces of averaged mEPSCs at P16 and P30, in ACSF (black) and after CTZ (gray). Scale bar: 5 pA, 10 ms. Summary graphs of mEPSC half-width before and after CTZ at P16 or P30 (D) and half-width increase (ΔHalf-width; E). (D) 2-way ANOVA, $F_{(23, 23)} = 2.217$, $p < 0.05$; ACSF vs. CTZ: $F_{(1,23)} = 521.7$, ****$p < 0.0001$; P16 vs. P30: $F_{(1,23)} = 0.109$, $p = 0.745$; interaction: $F_{(1,23)} = 1.213$, $p = 0.28$. (E) $T_{(23)} = 1.10$, $p = 0.28$. (A, B) Cumulative probability plots for AMPAR mEPSC amplitude before and after Cx614 at P16 (F) and P30 (G). (F) KS-Test, $p = 0.07$. (G) KS-Test, $p = 0.13$. (H) Sample traces of averaged mEPSCs at P16 and P30, in ACSF (black) and after Cx614 (gray). Scale bar: 5 pA, 10 ms. Summary graphs of mEPSC half-width before and after Cx614 at P16 or P30 (I) and half-width increase (ΔHalf-width; J). (I) 2-way ANOVA, $F_{(20,20)} = 1.283$, $p = 0.29$; ACSF vs. Cx614: $F_{(1,20)} = 9.654$, **$p < 0.01$; P16 vs. P30: $F_{(1,20)} = 6.384$, *$p < 0.05$; interaction: $F_{(1,20)} = 0.00133$, $p = 0.97$. (E) $T_{(20)} = 0.0591$, $p = 0.95$. (I, J) cumulative probability plots for AMPAR mEPSC amplitude. KS-Test, $p > 0.99$. (D) Sample traces of averaged mEPSCs at P16 and P30, in ACSF (black) and after aniracetam (Ani, gray). Scale bar: 5 pA, 10 ms. Summary graphs of mEPSC half-width before and after aniracetam at P16 or P30 (E) and half-width increase (ΔHalf-width; F). (E) 2-way ANOVA, $F_{(22, 22)} = 3.323$, $p < 0.05$; ACSF vs. Ani: $F_{(1,22)} = 130.1$, ****$p < 0.0001$; P16 vs. P30: $F_{(1,22)} = 7.292$, $p < 0.05$; interaction: $F_{(1,22)} = 0.025$, $p = 0.88$. (F) $T_{(22)} = 0.159$, $p = 0.88$.

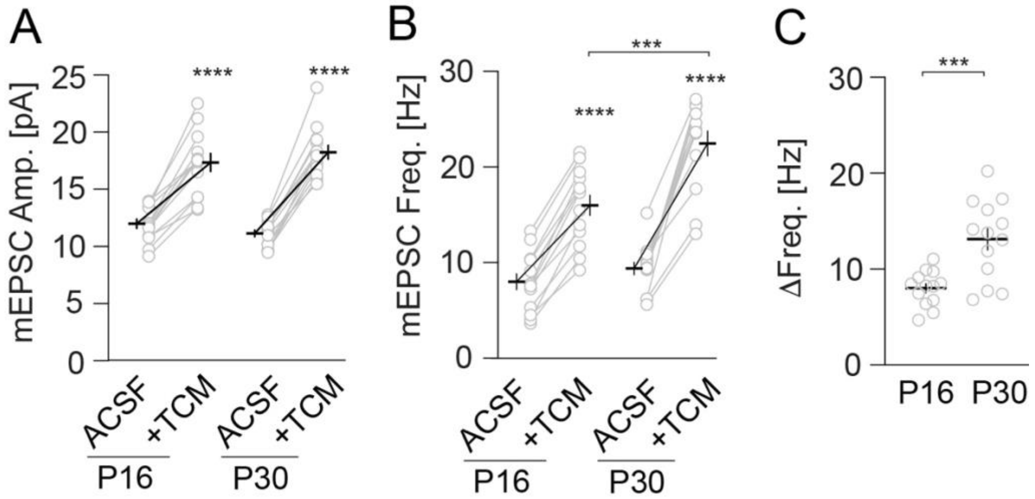

Fig. S6: Extended data for Figure 5.
Summary graph of AMPAR mEPSC amplitudes (A) and frequency (B) in the indicated experimental groups. (C) Summary graph of Δfrequency in the indicated experimental groups. Dots represent values of single neurons with the line representing the mean ± SEM. P16(13/8), P30(13/7). (A) 2-way ANOVA: $F_{(24, 24)} = 3.572$, $p < 0.01$; ACSF vs. TCM: $F_{(1, 24)} = 256.5$, ****$p < 0.0001$; P16 vs. P30: $F_{(1,24)} = 0.0317$, $p = 0.86$; interaction: $F_{(1,24)} = 5.557$, $p < 0.05$; TCM P16 vs. TCM P30: $p_{adj} = 0.86$. (B) 2-way ANOVA: $F_{(24, 24)} = 4.03$, $p < 0.001$; ACSF vs. TCM: $F_{(1, 24)} = 293.0$, ****$p < 0.0001$; P16 vs. P30: $F_{(1,24)} = 8.023$, $p < 0.01$; interaction: $F_{(1,24)} = 15.49$, $p < 0.001$. (C) $T_{(24)} = 3.935$, $p < 0.001$.

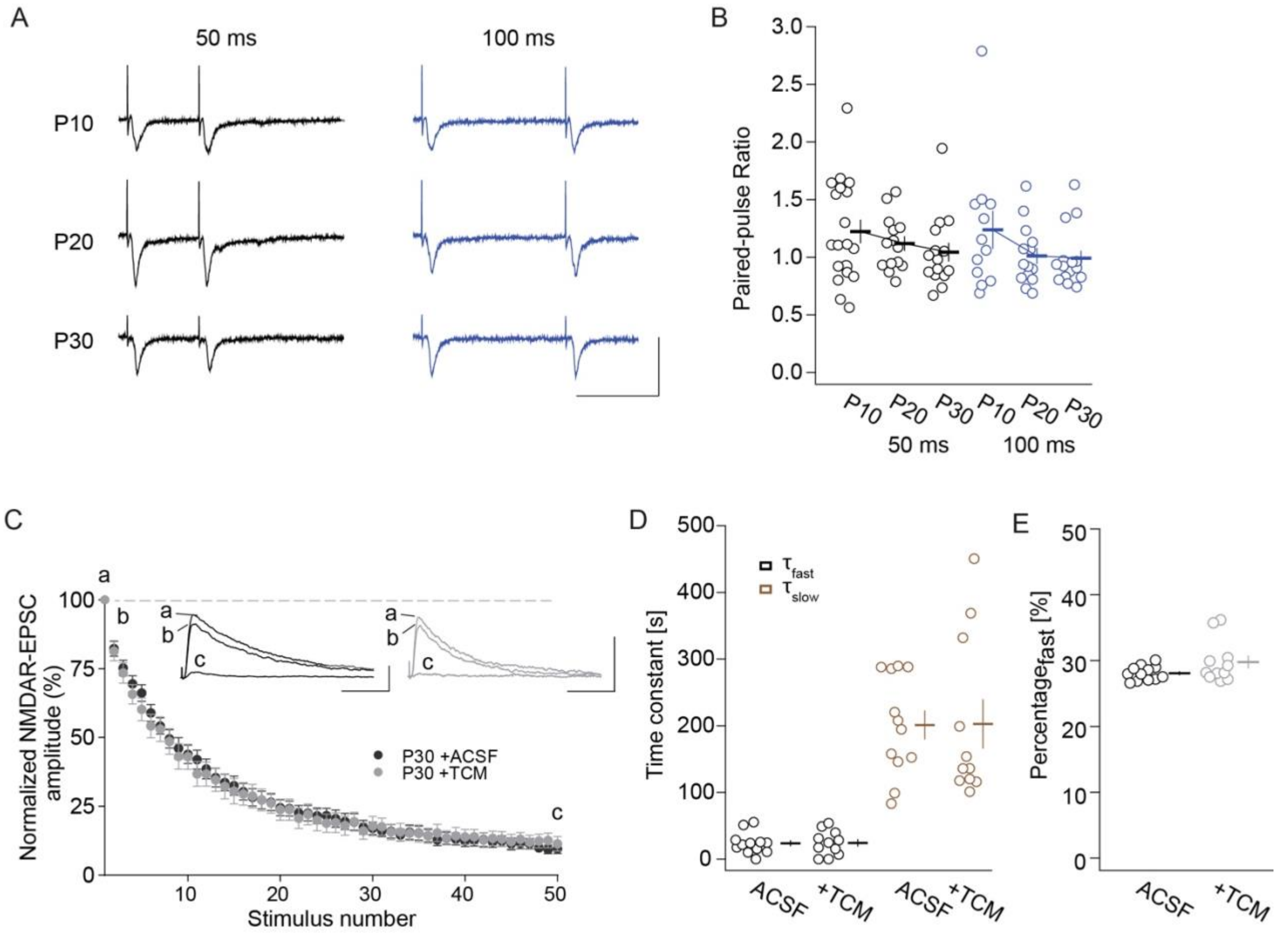

Fig. S7: Paired pulse ratio of AMPAR EPSCs were not affected by development and TCM. Sample traces (A) and summary graph (B) of paired pulse ratio (PPR) at the interval of 50 ms and 100 ms were recorded in V1 L4-L2/3 synapses from P10 to P30. PPR of individual neurons depicted as circles and average as bar with SEM. Scale bar: 50 ms and 200 pA. (B) P10 50 ms, (19/5), 100 ms (12/5), P14 (14/4), P30 (15/3). 50 ms: 1-way ANOVA, $F_{(2,45)} = 1.093$, $p = 0.34$. 100 ms: 1-way ANOVA, $F_{(2,38)} = 1.691$, $p = 0.20$. (C) Graph of averaged NMDAR response amplitude, normalized to the first response, versus stimulus number in the presence of the use-dependent NMDAR channel blocker MK-801. Inset: Sample traces showing progressive blockage of NMDAR currents in the presence of MK-801. Scale bar: 50 ms and 500 pA. (D) Time constants of the fast and slow (τ_{fast} and τ_{slow}) component and (E) percentage of fast component based on double exponential fit. (D) P30 (12/3), P30 +TCM (11/3). τ_{fast}: $T_{(21)} = 0.062$, $p = 0.95$. τ_{slow}: $T_{(21)} = 0.042$, $p = 0.97$. (E) $T_{(21)} = 1.702$, $p = 0.10$.

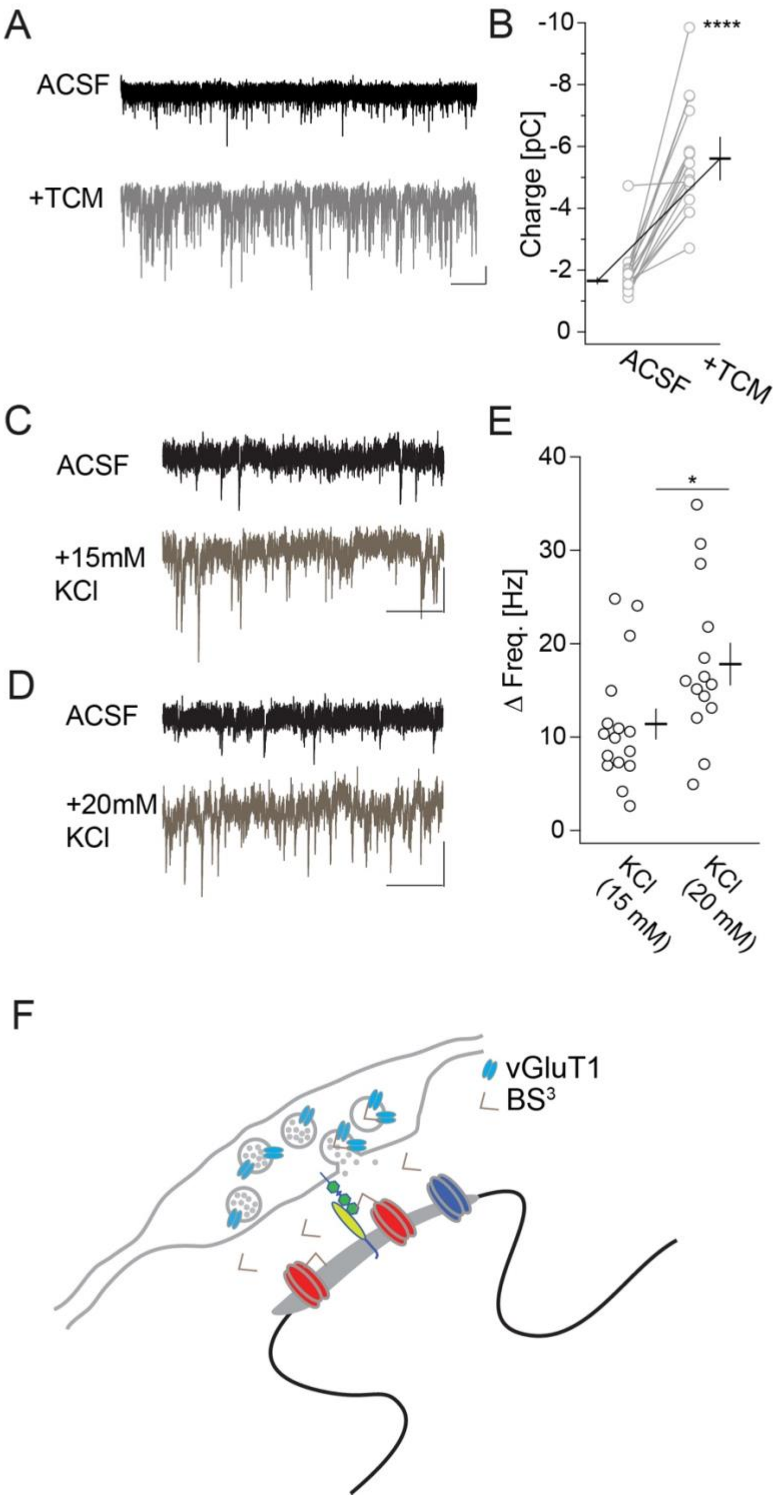

Fig. S8: Extended data for Figure 8.
(A, B) mEPSC were recorded with a V_h=-60mV. Sample traces in ACSF or with TCM (A) and summary graph of charge transfer before and after TCM (B). Scale bar: 1s, 10pA. (16/3); $T_{(15)}$=7.9, ****p<0.0001. (G-I) Sample traces in ACSF before and after 15mM (G) or 20mM KCl (H), and summary graph of charge increase after KCl (I). Scale bar: 200ms and 20pA. 15mM KCl (16/5), 20mM KCl (14/4); $T_{(28)}$=2.311, *p<0.05. (J) Experimental scheme illustrating the crosslinking of plasma membrane proteins with BS^3 to measure synaptic vesicle exocytosis.

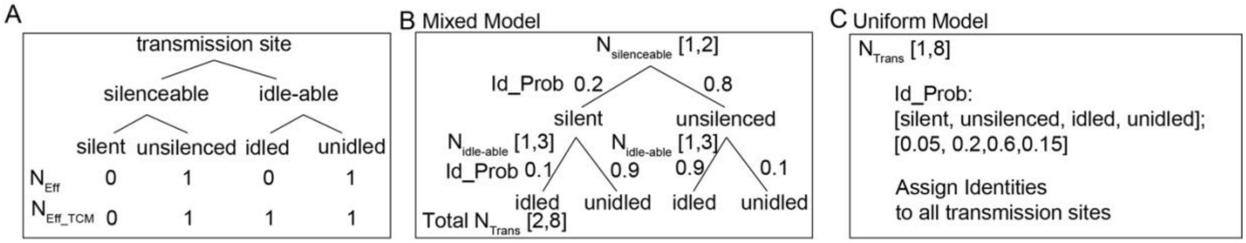

Fig. S9: Simulation Schematics.
(A) Identities of transmission sites, and the number of effective transmission site (N_{Eff}) associated with the identities. (B) The simulation of the spine head under the mixed model of transmission sites. 1-2 silenceable transmission sites are generated for each spine head with 20% probability as a silent site, and 80% probability as an unsilenced site. For each silenceable transmission site, 1-3 idle-able transmission sites are generated with it. To account for the relationship between idling and unsilencing, the probabilities of idled versus unidled transmission sites were set to 10% and 90% when associated with a silent site; and 90% versus 10% when associated with an unsilenced site. The total number of transmission sites per spine is 2-8. (C) The simulation of the spine head under the uniform model of transmission sites. The total number of transmission sites on each spine is set between 1 and 8. The probability of the identities of the transmission sites are set as 5%, 20%, 60% and 15% for silent silenceable, unsilenced silenceable, idled idle-able, unidled idle-able. The selected identity is assigned to all transmission sites on each spine head.

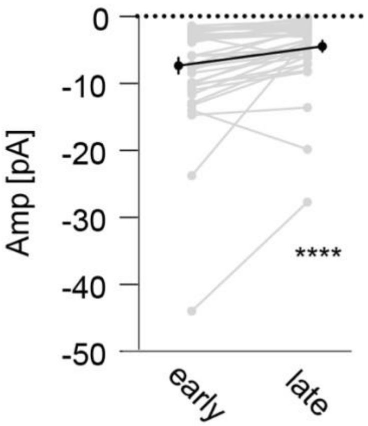

Fig. S10: Properties of two-photon uncaging of MNI-glutamate on visually identified spines.

Summary graph of uEPSCs amplitudes induced by two-photon uncaging of MNI-glutamate onto single spine head at the baseline condition (black) and repeated on the same spines after 5-10 min to assess stability of responses. Paired T-test, n/m: 72/5, $T_{(71)} = 6.99$, **** $p < 0.0001$.

Table S1: Gamma fit parameters for P16 and P30 neurons in ACSF and TCM

P16	ACSF								TCM							
	$\Gamma(\alpha,\beta)$		$\kappa * \Gamma_1(\alpha_1,\beta_1)+(1-\kappa) * \Gamma_2(\alpha_2,\beta_2)$					*p-value*	$\Gamma_T(\alpha_T,\beta_T)$		$\kappa_T * \Gamma_{1T}(\alpha_1,\beta_{1T})+(1-\kappa T) * \Gamma_{2T}(\alpha_2,\beta_{2T})$					*p-value*
			$\Gamma_1(\alpha_1,\beta_1)$		$\Gamma_2(\alpha_2,\beta_2)$		κ				$\Gamma_{1T}(\alpha_1,\beta_{1T})$		$\Gamma_{2T}(\alpha_2,\beta_{2T})$		κ_T	
	α	β	α_1	β_1	α_2	β_2			α_T	β_T	α_1	β_{1T}	α_2	β_{2T}		
1	8.13	1.34	9.59	1.52	23.11	0.38	0.36	0.00	5.54	2.39	9.59	1.84	23.11	0.40	0.48	0.00
2	6.92	1.68	8.53	1.81	21.84	0.41	0.41	0.00	5.29	3.12	8.53	2.63	21.84	0.53	0.45	0.00
3	9.90	1.22	6.91	2.64	19.61	0.56	0.15	0.00	7.18	2.73	6.91	3.24	19.61	0.78	0.60	0.00
4	4.23	3.31	14.09	1.79	16.00	0.63	0.26	0.00	4.53	3.85	14.09	2.01	16.00	0.80	0.30	0.00
5	5.02	2.67	4.81	4.98	13.96	0.79	0.19	0.00	4.61	3.77	4.81	4.66	13.96	0.88	0.50	0.00
6	14.76	0.66	11.52	1.18	26.18	0.35	0.15	0.00	7.71	1.86	11.52	1.47	26.18	0.39	0.61	0.00
7	8.38	1.43	9.42	1.57	19.11	0.51	0.46	0.00	5.10	3.58	9.42	2.51	19.11	0.64	0.53	0.00
8	5.71	2.34	6.94	2.50	19.81	0.49	0.48	0.00	4.01	5.62	6.94	4.21	19.81	0.69	0.57	0.00
9	7.23	1.64	8.50	1.86	17.85	0.53	0.37	0.00	4.06	4.35	8.50	3.06	17.85	0.66	0.42	0.00
10	7.81	1.49	8.47	1.78	17.90	0.53	0.38	0.00	4.89	3.59	8.47	2.77	17.90	0.66	0.49	0.00
11	5.79	2.40	6.46	2.84	16.10	0.66	0.42	0.00	4.25	4.99	6.46	4.17	16.10	0.84	0.57	0.00
12	15.18	0.60	10.57	1.24	24.92	0.35	0.12	0.00	8.83	1.52	10.57	1.45	24.92	0.42	0.60	0.00
13	10.00	1.08	9.96	1.42	22.57	0.41	0.30	0.00	8.09	1.77	9.96	1.80	22.57	0.51	0.44	0.00

P30	ACSF								TCM							
	$\Gamma(\alpha,\beta)$		$\kappa * \Gamma_1(\alpha_1,\beta_1)+(1-\kappa) * \Gamma_2(\alpha_2,\beta_2)$					*p-value*	$\Gamma_T(\alpha_T,\beta_T)$		$\kappa_T * \Gamma_{1T}(\alpha_1,\beta_{1T})+(1-\kappa T) * \Gamma_{2T}(\alpha_2,\beta_{2T})$					*p-value*
			$\Gamma_1(\alpha_1,\beta_1)$		$\Gamma_2(\alpha_2,\beta_2)$		κ				$\Gamma_{1T}(\alpha_1,\beta_{1T})$		$\Gamma_{2T}(\alpha_2,\beta_{2T})$		κ_T	
	α	β	α_1	β_1	α_2	β_2			α_T	β_T	α_1	β_{1T}	α_2	β_{2T}		
1	10.86	1.11	5.49	3.78	18.01	0.63	0.07	0.00	6.87	2.62	5.49	3.61	18.01	0.82	0.64	0.00
2	11.15	1.02	9.69	1.58	20.02	0.51	0.21	0.00	6.14	2.81	9.69	2.23	20.02	0.62	0.53	0.00
3	13.21	0.77	14.07	0.95	24.86	0.37	0.24	0.00	6.11	2.80	14.07	1.66	24.86	0.50	0.42	0.00
4	14.75	0.65	13.20	0.92	22.35	0.40	0.20	0.00	7.05	2.39	13.20	1.68	22.35	0.57	0.43	0.00
5	12.29	0.78	10.40	1.20	20.78	0.42	0.22	0.00	6.99	2.26	10.40	1.90	20.78	0.57	0.50	0.00
6	9.08	1.11	6.83	2.58	17.84	0.52	0.10	0.00	5.28	3.06	6.83	3.34	17.84	0.70	0.36	0.00
7	7.27	1.74	6.13	3.12	18.35	0.59	0.22	0.00	3.80	6.27	6.13	4.93	18.35	0.77	0.60	0.00
8	9.93	1.17	8.40	2.05	16.11	0.66	0.14	0.00	4.31	4.40	8.40	3.12	16.11	0.79	0.47	0.00
9	13.47	0.70	7.80	1.99	18.60	0.49	0.05	0.00	6.84	2.66	7.80	2.78	18.60	0.75	0.55	0.00
10	9.19	1.24	6.28	2.84	15.83	0.67	0.12	0.00	6.17	3.11	6.28	3.73	15.83	0.94	0.50	0.00
11	11.06	1.13	11.48	1.49	18.28	0.62	0.20	0.00	6.29	3.23	11.48	2.27	18.28	0.80	0.49	0.00
12	8.53	1.37	7.51	2.25	15.95	0.65	0.20	0.00	5.37	3.33	7.51	2.95	15.95	0.78	0.56	0.00
13	9.40	1.16	8.91	1.62	20.47	0.46	0.29	0.00	6.35	2.42	8.91	2.20	20.47	0.57	0.47	0.00

Table S2: Key resources are listed.

Antibodies

Name	RRID	clone	source
vGluT1	RRID:AB_2877383	N28/9	NeuroMab
GluA2	RRID:AB_2877267	L21/32	NeuroMab
Actin	RRID:AB_2537000	AC-40	Thermo Fisher

Cell lines

Name	ID	source
293T	CRL-3216	ATCC

Mouse lines

Name	RRID	source
C57Bl/6J	IMSR_JAX:000664	In house breeding

AAV

Name	source
AAV-sh95-GFP	PMID: 26015564
AAV-shLc-GFP	PMID: 26015564
